# SensorFRET: A Standardless Approach to Measuring Pixel-based Spectral Bleed-through and FRET Efficiency using Spectral Imaging

**DOI:** 10.1038/s41598-017-15411-8

**Published:** 2017-11-15

**Authors:** Paul T. Arsenovic, Carl R. Mayer, Daniel E. Conway

**Affiliations:** Virginia Commonwealth University, Department of Biomedical Engineering, Richmond, VA 23284 USA

## Abstract

Fluorescence microscopy of FRET-based biosensors allow nanoscale interactions to be probed in living cells. This paper describes a novel approach to spectrally resolved fluorescence microscopy, termed sensorFRET, that enables quantitative measurement of FRET efficiency. This approach is an improvement on existing methods (FLIM, sRET, luxFRET, pFRET), as it does not require single fluorophore standards to be measured with every experiment and the acquisition is intensity independent, allowing the laser power to be optimized for varying levels of fluorophore expression. Additionally, it was found that all spectral based methods, including sensorFRET, fail at specific fluorophore-excitation wavelength combinations. These combinations can be determined a priori using sensorFRET, whereas other methods would give no indication of inaccuracies. This method was thoroughly validated and compared to existing methods using simulated spectra, Fluorescein and TAMRA dye mixtures as a zero FRET control, and Cerulean-Venus FRET standards as positive FRET controls. Simulations also provided a means of quantifying the uncertainty in each measurement by relating the fit residual of noisy spectra to the standard deviation of the measured FRET efficiency. As an example application, Teal-Venus force sensitive biosensors integrated into E-cadherin were used to resolve piconewton scale forces along different parts of an individual cell junction.

## Introduction

Forster Resonant Energy Transfer (FRET) is an invaluable tool for the nano-scale examination of a variety of interactions in live-cells. FRET arises from the non-radiative transfer of energy between two fluorophores termed donor and acceptor^1^. The FRET transfer efficiency between the donor and acceptor fluorophores is extremely sensitive to the distance between the fluorophores (*r* ^6^), allowing this technique to resolve sub nanometer scale changes in the fluorophore separation. The engineering of a variety of fluorescent proteins has led to the increased use of genetically-encoded FRET-based biosensors in which the donor and acceptor molecules are held together by peptide linkers^2,3^. These sensors are capable of measuring various intra-cellular processes that occur on a scale between 1–10 nanometers, well below the diffraction-limit of optical microscopes. A large number of FRET biosensors have been engineered to study protein cleavage, protein conformation changes, local redox and pH sensing, and determining the mechanical load on force bearing proteins^4^.

Genetically-encoded, unimolecular FRET biosensors are particularly useful because they are generally less-toxic than cellular dyes and they can be directed to specific regions or organelles in the cell^5^. Measurements of FRET in unimolecular biosensors are simplified by the fact that donor and acceptor fluorophores are expressed in the same molecule^6^. A simple ratio image of the resolved emissions of the donor and acceptor yields a non-linear FRET index that is correlated, but not equal to the FRET transfer efficiency^7^. This form of FRET imaging monitors the sensitized emission of the acceptor and quenched emission of the donor, a direct measure of the transfer of energy between the donor and acceptor molecules^8^.

Measuring sensitized-emission FRET (SeFRET) is a common technique to monitor unimolecular sensors due to the ease of capturing ratio images and the speed at which they can be acquired^9^. For studies that require increased temporal resolution, measurements of SeFRET are preferred since photo toxicity and image acquisition time is minimal when compared to photo-bleaching or fluorescent lifetime imaging (FLIM) respectively^10^. While uncorrected ratio-images can be used to monitor relative changes in FRET, they cannot be used to quantitatively measure the transfer efficiency of FRET. The transfer efficiency is generally the most useful parameter in FRET experiments because it is independent of the measuring equipment and can be used to estimate the distance between fluorophores in the sensor^11^. Knowing the true FRET efficiency is particularly pertinent for FRET-force probes since the force calibrations for these sensors are reported in units of FRET transfer efficiency^12–16^. Quantitative determination of the FRET efficiency requires a variety of controls to account for the experimental variables such as photobleaching/toxicity, cellular environment effects, cell line dependent effects, autofluorescence, and additional fluorescent labels used. However, one of the most difficult challenges with determining the FRET efficiency using SeFRET is accurately removing spectral bleed-through (also known as cross-talk)^17^. Using current methods, spectral bleed-through from the direct excitation of the acceptor fluorophore cannot be removed from SeFRET images without calibration measurements requiring donor-only or acceptor-only control samples and the implementation of correction algorithms after image capture^17–20^. Typically it it necessary to take these calibration measurements along with every experimental data set, as the corrections are power dependent and will therefore vary as the laser output changes over time^21^.

A rapid and simple method to measure spectral bleed-through in experimental samples that does not require control samples or complicated corrections would make quantitative seFRET more attractive to researchers that need fast and quantitative measurements of FRET efficiency. In this work, we present a novel method to quantitatively measure FRET efficiency using SeFRET that does not require a lengthy calibration and the uncertainty of the FRET efficiency can be determined on a per-pixel basis. Since this method relies on a curve-fitting approach, the signal to noise ratio (SNR) can be estimated by computing normalized residuals on each image pixel and then correlated to the error in the FRET efficiency estimate. Using the normalized residual error as an SNR metric, SensorFRET images can be thresholded by the estimated uncertainty in each pixel depending on the precision requirements of the experiment. This method can be implemented on any type of microscope equipped with at least two excitation wavelengths and a detector with spectral resolution.

The acquisition routine of this method is substantially easier to implement than other established spectral imaging methods. For SensorFRET as well as other published spectral methods (luxFRET, sRET, and pFRET)^17,19,20^, images of the same region must be acquired using two different excitation wavelengths. SensorFRET is unique from other spectral methods since no additional calibration images are required for the bleed through correction and the laser power and gain settings for each image may be adjusted independently in order to achieve the best imaging conditions. This is in stark contrast to the calibration requirements needed for the other methods, which require one or more single fluorophore standards to be imaged prior to every experiment and the laser power/gain settings must be maintained between the calibration and experiment. LuxFRET requires imaging of two cell cultures expressing only donor or acceptor flurophores, sRET requires imaging of two solutions with known concentrations of the donor or acceptor fluorophore, and pFRET requires imaging of a single cell culture expressing only the acceptor fluorophore, but has additional restrictions on which excitation wavelengths can be used^17,19,20^. SensorFRET, which only requires a spectral detector and two excitation sources (which are more readily available than FLIM acquisition equipment) and eliminates the additional complication, time, and expense associated with calibration sample preparation, provides an easy and accessible method for accurately determining the FRET efficiency, enabling FRET analysis to be utilized by a broader range of the research community.

## Results and Discussion

### Analysis Approach

For the purposes of our analysis, the fluorescent output of a FRET construct, ${F}_{DA}({\lambda }_{em},{\lambda }_{ex})$, is a function of emission wavelength, ${\lambda }_{em}$, and excitation wavelength, ${\lambda }_{ex}$, and can be thought of as the linear combination of five components:

$$\begin{array}{c}{F}_{DA}({\lambda }_{em},{\lambda }_{ex})={\rm{FRET}}\,{\rm{Donor}}+{\rm{FRET}}\,{\rm{Acceptor}}+{\rm{FRET}}\,{\rm{Acceptor}}\,{\rm{Direct}}\,{\rm{Excitation}}\,+\\ \quad \,\,\,\,\,\,\,\,\,\,\,\,\,\,\,\,\,\,\,\,\,\,\,{\rm{Unpaired}}\,{\rm{Donor}}+{\rm{Unpaired}}\,{\rm{Acceptor}}\end{array}$$

Since both the donor and acceptor are synthesized simultaneously, the use of uni- or bi-molecular FRET sensors greatly reduces the effect of unpaired fluorophores on the fluorescence emission. Therefore, the main challenge remaining in accurately determining the FRET efficiency is separating the acceptor emission due to FRET from the acceptor emission due to direct excitation (the acceptor fluorescence in the absence of the donor). It should be noted this analysis is valid for unpaired molecules where there is excess acceptor, however invalid for FRET experiments with unpaired donors (see Supplemental Note 1 for a detailed analysis). Since both the FRET Acceptor and FRET Acceptor Direct Excitation have the same emission spectra, linear unmixing of a single spectra cannot separate the two contributions. SensorFRET takes advantage of the fact that the acceptor emission due to FRET has a different dependence on excitation wavelength than the acceptor emission due to direct excitation. This allows the calculation and removal of the acceptor direct excitation term using images of the same region of interest at two different excitation wavelengths, 1 and 2.

**Figure 1:**
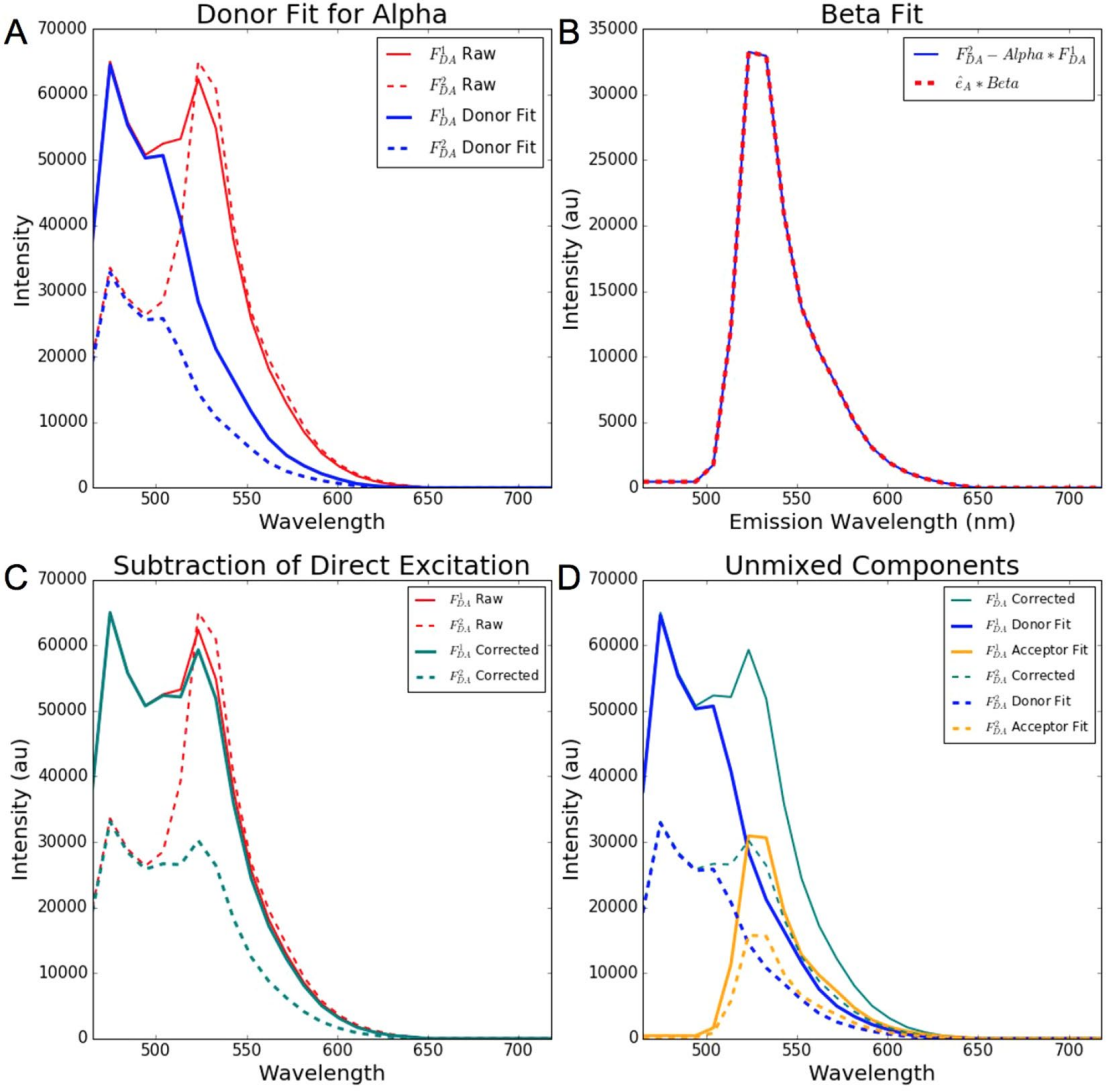
Fitting procedures for calculating the FRET efficiency on simulated FRET spectra with 35% transfer efficiency. (**A**) Fitting of the donor contribution to determine *α*, (**B**) Fitting of the $\beta$ term, (**C**) Subtraction of the acceptor direct excitation, (**D**) Linear unmixing of the donor and acceptor components.

A full derivation of the SensorFRET approach is provided in the Supplementary Note 1. The results of this analysis show that there are only three key parameters needed to calculate and remove the FRET Acceptor Direct Excitation and the Unpaired Acceptor contributions. The first of these, *α*, is defined as:

1 $$\alpha \equiv \frac{{F}_{DA}(donor,{\lambda }_{ex2})}{{F}_{DA}(donor,{\lambda }_{ex1})}$$

where ${F}_{DA}(donor,{\lambda }_{exi})$ is the magnitude of the donor component of the FRET spectra at excitation wavelength *i*, determined through linear unmixing (Fig. 1A). The second parameter needed is $\beta$, which is defined as:

2 $$\beta \times {\hat{e}}_{A}\equiv {F}_{DA}({\lambda }_{em},{\lambda }_{ex2})-\alpha \times {F}_{DA}({\lambda }_{em},{\lambda }_{ex1})$$

where ${F}_{DA}({\lambda }_{em},{\lambda }_{exi})$ is the raw FRET spectra at excitation wavelength *i*, and $\beta$ is determined by least squares fitting the right hand side of equation (2) to the normalized acceptor emission shape, ${\hat{e}}_{A}$ (Fig. 1B). The final parameter required is *γ*, which is defined as:

3 $$\begin{array}{ll}\gamma  & \equiv \frac{{\varepsilon }_{D2}}{{\varepsilon }_{D1}}\times \frac{{\varepsilon }_{A1}}{{\varepsilon }_{A2}}\\  & =\frac{{\hat{\varepsilon }}_{D2}}{{\hat{\varepsilon }}_{D1}}\times \frac{{\hat{\varepsilon }}_{A1}}{{\hat{\varepsilon }}_{A2}}(from\,literature)\\  & or\\  & =\,\frac{{F}_{D}({\lambda }_{em},{\lambda }_{ex2})}{{F}_{D}({\lambda }_{em},{\lambda }_{ex1})}\times \frac{{F}_{A}({\lambda }_{em},{\lambda }_{ex1})}{{F}_{A}({\lambda }_{em},{\lambda }_{ex2})}(from\,single\,fluorophore\,cell\,cultures)\end{array}$$

where ${\varepsilon }_{Xi}$ is the molar extinction coefficient of fluorophore *X* at excitation wavelength *i*, ${\hat{\varepsilon }}_{Xi}$ is the value of the excitation spectra of fluorophore *X* at excitation wavelength *i*, and ${F}_{X}({\lambda }_{em},{\lambda }_{exi})$ is the raw spectra at excitation wavelength *i* from a cell expressing a single fluorophore *X*. This parameter can be determined from normalized excitation spectra which are readily available in the literature for commonly used fluoroscent proteins^22^ allowing the FRET efficiency to be determined without any of the standards needed by other spectral intensity approaches^17–20^. If excitation spectra are not available for the fluorophores, this parameter can also be determined experimentally from a one-time measurement of two cell cultures, one expressing only the donor fluorophore and the other expressing only the acceptor fluorophore (See Supplemental Figure S1). For the experimental determination of *γ*, it is important that the images taken at both excitation wavelengths are aligned spatially for a given cell culture and that the laser power and gain settings are maintained while imaging the donor-only and acceptor-only cell cultures with a given excitation wavelength (see Supplementary Note 1 for justification of this requirement). If *γ* is measured properly, it is independent of the instrumentation parameters and depends solely on the fluorophores in the sensor, the two excitation wavelengths used, and the local cellular environment.

With these three parameters, we are able to correct for the FRET Acceptor Direct Excitation and Unpaired Acceptor contributions at excitation wavelengths 1 and 2, as shown in Fig. 1C, according to the following equations:

4 $${F}_{DAcorr}({\lambda }_{em},{\lambda }_{ex1})={F}_{DA}({\lambda }_{em},{\lambda }_{ex1})-\frac{\beta }{\alpha ({\gamma }^{-1}-\mathrm{1)}}\times {\hat{e}}_{A}$$

5 $${F}_{DAcorr}({\lambda }_{em},{\lambda }_{ex2})={F}_{DA}({\lambda }_{em},{\lambda }_{ex1})-\frac{\beta }{1-\gamma }\times {\hat{e}}_{A}$$

Assuming the Unpaired Donor contribution is negligible, the corrected spectra can then be unmixed into FRET Donor and FRET Acceptor components using linear unmixing (Fig. 1D) and the FRET efficiency at excitation wavelength *i*, $E({\lambda }_{exi})$, can then be determined according to:

6 $$E({\lambda }_{exi})={(\frac{{F}_{DAcorr}(donor,{\lambda }_{exi})\ast {Q}_{A}}{{F}_{DAcorr}(acceptor,{\lambda }_{exi})\ast {Q}_{D}}+1)}^{-1}$$

where ${F}_{DAcorr}(donor,{\lambda }_{exi})$ is the magnitude of the donor component of the corrected spectra at excitation wavelength *i*, ${F}_{DAcorr}(acceptor,{\lambda }_{exi})$ is the magnitude of the acceptor component of the corrected spectra at excitation wavelength *i*, ${Q}_{A}$ is the quantum efficiency of the acceptor fluorophore, and ${Q}_{D}$ is the quantum efficiency of the donor fluorophore (the values of which are taken from literature).

### Simulation

In order to validate this method and compare it to the sRET^20^, luxFRET^19^, and pFRET^17^ spectral imaging methods, FRET spectra with a pre-determined FRET efficiency (35%) were generated from excitation and emission spectra for Cerulean and Venus fluorophores available in the literature^23^, as detailed in the methods section and shown in Supplementary Figure S2. Wavelengths 1 and 2 were chosen to be 405 and 458 nm, corresponding to the laser lines used in the subsequent experimental section. The *α* and $\beta$ terms were determined using the fits shown in Fig. 1A,B, respectively, while the *γ* term was calculated from the literature excitation spectra to have a value of 0.045. sRET, luxFRET, and pFRET algorithms were implemented according to their original references^17,19,20^. Under these ideal and noiseless conditions, all 4 approaches yielded a FRET efficiency of exactly 35%, corresponding to the pre-determined simulated value within floating point precision.

**Figure 2:**
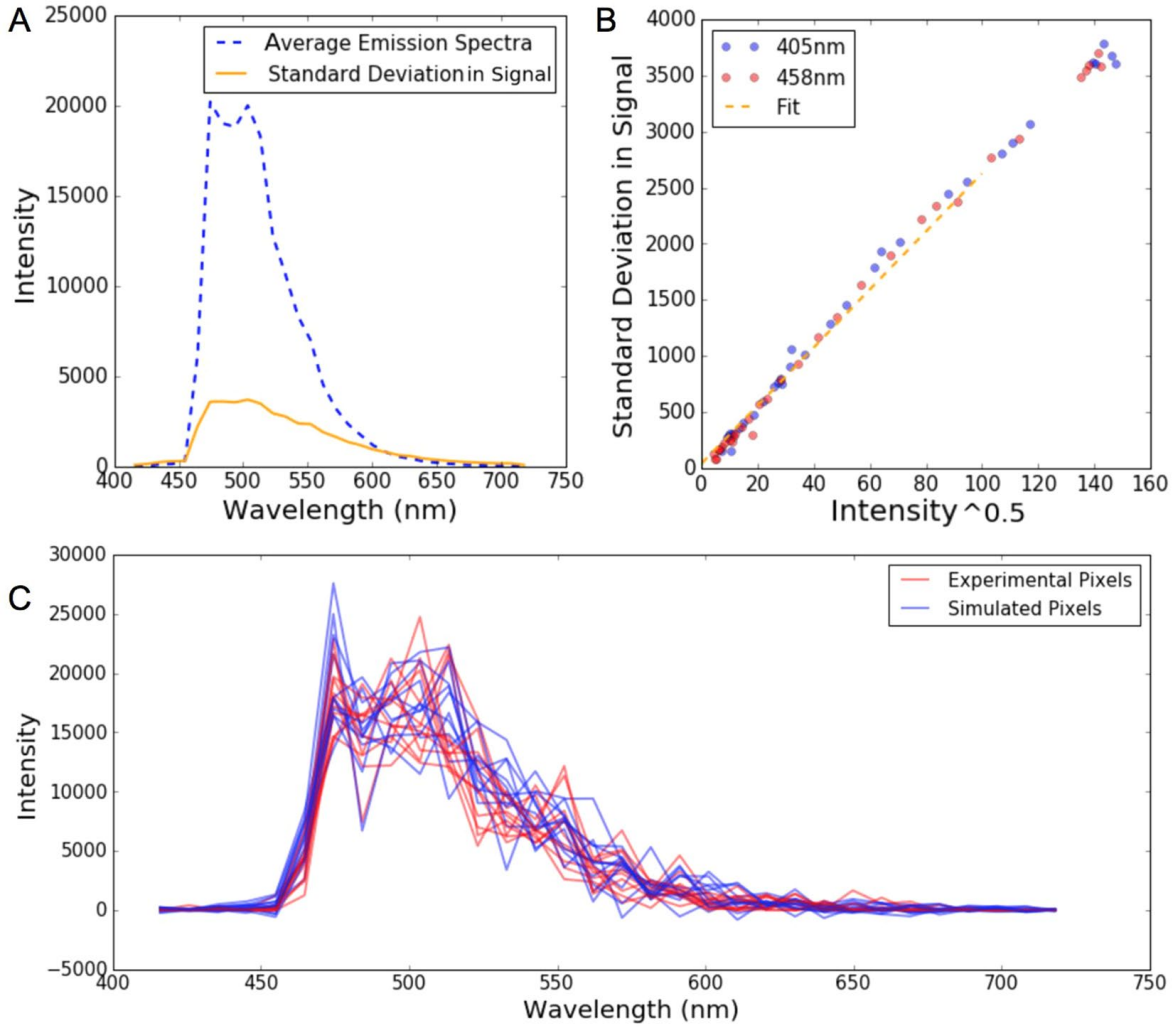
Determination of an accurate noise model. (**A**) Signal intensity and standard deviation as a function of emission wavelength in Cerulean-Traf-Amber sample. (**B**) Signal standard deviation vs the square root of the intensity. (**C**) comparison of experimental and simulated noisy pixels.

Besides confirming the mathematical approach of SensorFRET, another goal of these simulations was to characterize the noise dependence of this method and compare it to the other spectral intensity based methods. In order to add realistic noise to simulated pixels of a known FRET efficiency, the variability in the signal was characterized as a function of spectra amplitude. To quantify this variability, the difference was taken between each single pixel spectra and the average emission shape (scaled to have equivalent intensity). In practice this scaling is determined by a least-squares fitting of the average emission shape with each single noisy pixel spectra. Then the standard deviation of these differences, which we call the standard deviation in signal, was calculated for each emission channel (Fig. 2A). The standard deviation in signal is linearly dependent on the square root of the signal intensity (Fig. 2B). This type of noise dependence is known as shot or Poisson noise and is characteristic of the photomultiplier tube (PMT) detector^24^. Using the fit parameters from Fig. 2B, realistic noise can be added to simulated spectra making them nearly indistinguishable from experimentally observed spectra from individual pixels (Fig. 2C).

**Figure 3:**
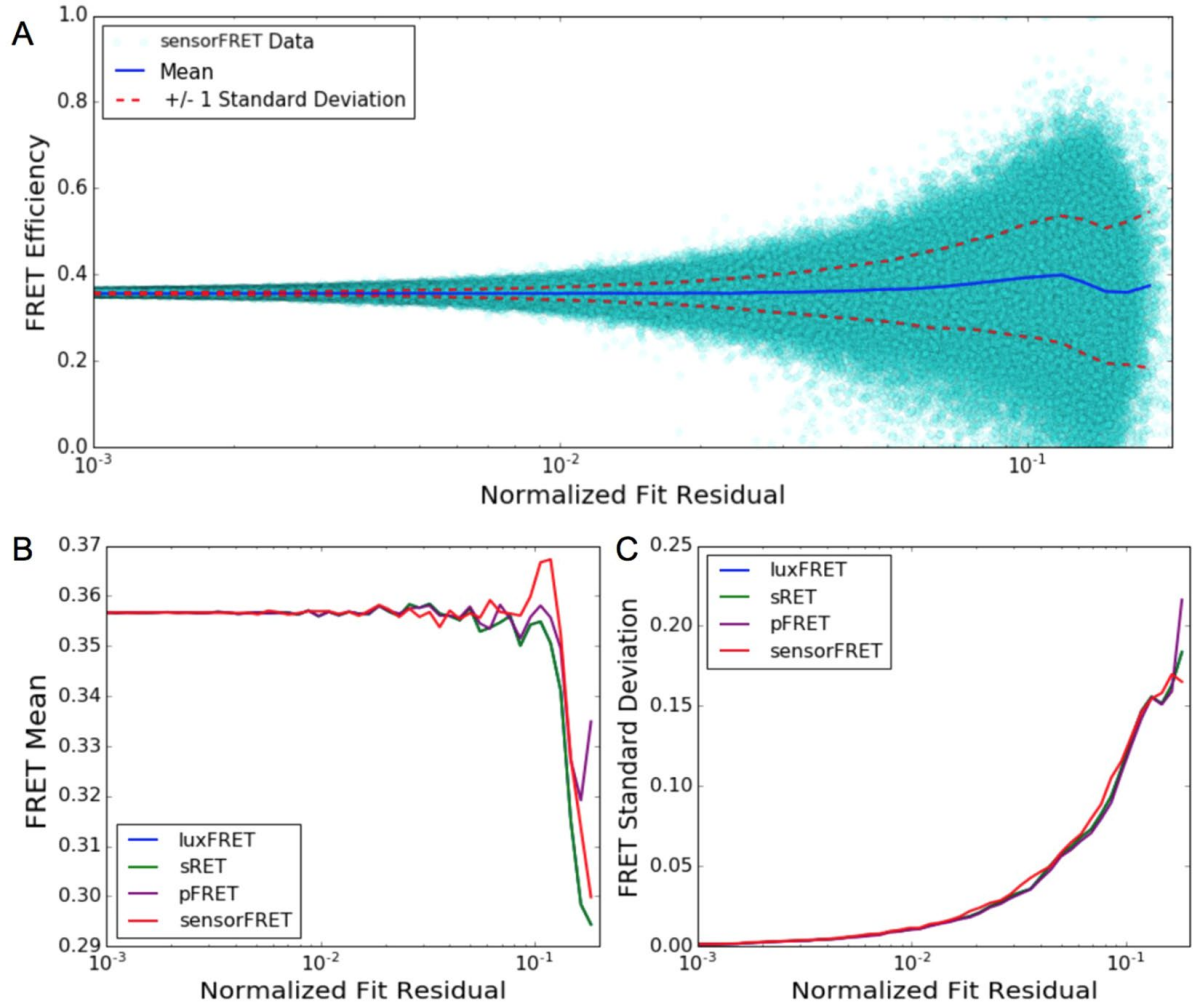
Comparison of noise tolerance of the SensorFRET method to sFRET, luxFRET, and pFRET. No appreciable difference between the methods is observed. (**A**) FRET efficiency vs normalized fit residual for 60000 simulated pixels (3000 pixels at 20 signal to noise ratios). (**B**) Median estimate for each of the methods as a function of residual. (**C**) Standard deviation for each of the methods as a function of residual.

By simulating pixels covering a range of signal to noise ratios, we determined the expected standard deviation in the measured FRET efficiency as a function of the normalized spectra fit residual (Fig. 3A). The normalized fit residual is used as a measurable metric for the signal to noise ratio (SNR) in the spectra because it can be calculated on a per-pixel basis. For sensorFRET, this approach is preferable to characterizing the SNR as a function of absolute intensity by (apparent photons) or by (photon conversion factor) at a constant set of detector settings, as described by Woehler *et al*.^25^ for the luxFRET approach and Hoppe *et al*.^26^ for N-Way FRET. This is because SensorFRET, in contrast to luxFRET, can use spectra acquired at any laser and detector settings and it is therefore preferable to use a metric for the SNR that is also independent of these parameters, such as the normalized residual, rather than try to characterize the SNR vs intensity behavior for all possible detector settings. It should be noted that these simulations depend on the fluorophore emission shape, excitation wavelengths, and the estimated gamma parameter. In general, any deviation in these inputs will change the estimated FRET standard deviation at a given SNR. The same simulated input spectra used to form Fig. 3A was analyzed using the sRET, luxFRET and pFRET methods (while using perfect, noiseless calibration spectra required for each analysis approach) to obtain analogous estimations of the mean and standard deviation of the FRET as a function of residual. These are plotted in Fig. 3B,C, which shows no appreciable difference in the noise tolerance between any of the methods. In practice SensorFRET will likely outperform the compared methods since the laser excitation and detector gain can be adjusted on an image-by-image basis. Most biological samples exhibit large variance in the expression level of FRET sensors^27^, therefore optimizing the excitation power and gain for a given measurement will improve the signal-to-noise ratio for observed concentrations that deviate from calibration measurements.

We have also generated  a 5D array with simulated Cerulean-Venus FRET spectra at 405 and 458 that can be used for benchmark testing FRET analysis methodologies, available at https://doi.org/10.6084/m9.figshare.5573542.v1. The dataset contains a total of 3 million simulated spectra across a range of FRET efficiencies and signal to noise ratios (SNR) as well as all of the calibration spectra that would be required to analyze the data using sensorFRET, luxFRET, or sRET. The data is packaged in both matlab and python format and organized as described in the supplemental material.

### Validation Using Dye Solutions

While the simulations in the preceeding section show that SensorFRET and published spectral imaging methods (sRET, LuxFRET, and pFRET) are mathematically correct, they do not measure the accuracy of the algorithm under conditions where instrumentation can bias the measurement. The FRET spectra model outlined in the Supplemental Equation S3 could be incomplete and fail under realistic experimental conditions. To test the validity of the FRET spectra model and the effect of excitation pairing on SensorFRET, experiments using a soluble FRET-compatible dye pair was conducted using both a spectrofluorometer and FLIM microscope.

**Figure 4:**
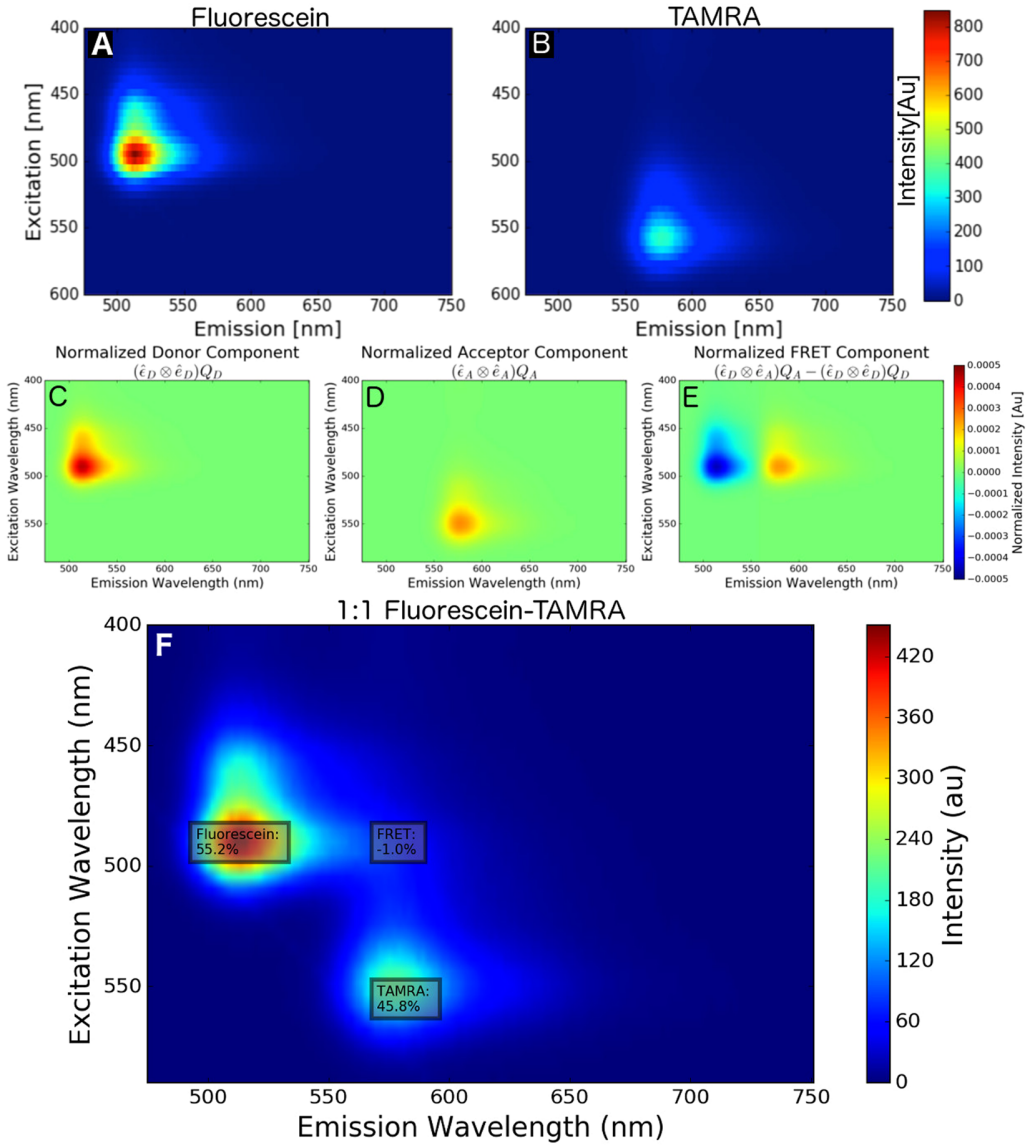
Measured EEM of Fluorescein and TAMRA solutions at 2 $\mu$M concentration in PBS, (**A**) and (**B**) respectively. Donor, Acceptor, and FRET components used for linear unmixing were generated from the normalized excitation and emission spectra as shown in (**C**), (**D**), and (**E**), respectively. Panel F shows the EEM of a 1:1 mixture of the Fluorescein and TAMRA solutions with the relative magnitudes of the fitting components superimposed (Donor: 55.2%, Acceptor: 45.8%, FRET: −1.0%)

We chose Fluorescein and TAMRA as a FRET compatible dye pair. These fluorescent dyes are frequently used as standard reference dyes and their spectral properties are sufficient for energy transfer given the fluorophores are separated by 1–10 nm. Specifically, there is a sufficient overlap of the Fluorescein emission spectrum with the excitation spectrum of TAMRA (Supplementary Figure S3). Furthermore, the spectra and lifetime of these dyes are well characterized, therefore any instrumental or methodological errors may be readily identified in measurements. To determine the FRET between dilute mixtures of Fluorescein and TAMRA, first the excitation-emission matricies (EEM) of single-dye solutions (at 1 $\mu$M concentration) were characterized with a spectrofluorometer (Fig. 4A,B). Each of the Donor, Acceptor, and FRET EEM (Fig. 4C–E) used for the fitting process were derived by taking the outer product of a normalized excitation spectra and normalized emission spectra, ie. Donor ${\rm{EEM}}=({\hat{\varepsilon }}_{D}\otimes {\hat{e}}_{D}){Q}_{D}$, Acceptor ${\rm{EEM}}=({\hat{\varepsilon }}_{A}\otimes {\hat{e}}_{A}){Q}_{A}$, and the FRET ${\rm{EEM}}=({\hat{\varepsilon }}_{D}\otimes {\hat{e}}_{A}){Q}_{A}-({\hat{\varepsilon }}_{D}\otimes {\hat{e}}_{D}){Q}_{D}$. When these components are used to fit an experimental EEM, the ratio of the magnitude of the FRET EEM to the magnitude of the Donor EEM yields the FRET efficiency.

Using linear unmixing, the normalized EEM shown in Fig. 4C–E were used to determine the relative magnitude of each component in a solution containing a mixture of Fluorescein and TAMRA (both at 1 $\mu$M concentration) shown in Fig. 4F. The relative magnitudes of the Donor, FRET, and Acceptor contributions to the signal were 55.2%, 45.8%, and −1.0%, respectively. As the magnitude of the FRET component is approximately 0%, this shows that there is insignificant energy transfer between the Fluorescein and TAMRA, as expected for a dilute solution.

To independently confirm the dye pair had zero FRET, the fluorescent lifetime of Fluorescein and the dye mixture (Fluorescein + TAMRA) were measured using time-correlated single photon counting (TCSPC). To ensure calibration of the TCSPC equipment, Fluorescein was diluted to 50 $\mu$M in sodium borate and measured as a droplet on a glass slide. The published lifetime for Fluorescein at high pH is 4.1 ns^28–30^, which agrees with our measurement of 4.19 or 4.13 ns (for global or 10 × 10 binned data respectively: see Supplemental Figure S4).

**Figure 5:**
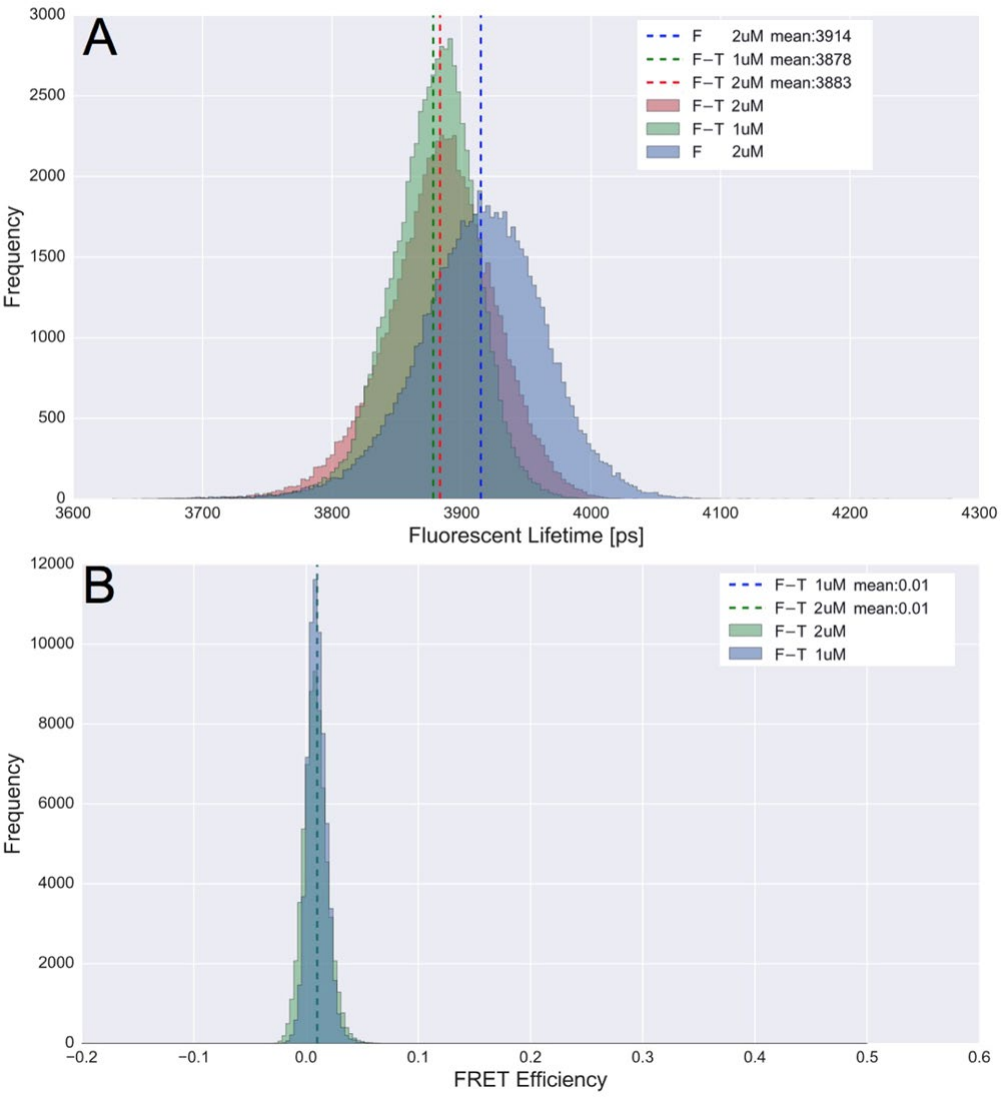
Fluorescein and TAMRA Fluorescent Lifetime and FRET Efficiency Distributions: Lifetimes were captured on an inverted microscope using time-correlated single photon counting (TCSCP). (**A**) Fluorescent lifetime distributions for Fluorescein (F-Blue), Fluorescein and TAMRA at 1 $\mu$M (F-T-1uM-Green) and Fluorescein and TAMRA at 2 $\mu$M (F-T-2uM-Red). Means of the lifetime distributions are shown as vertical dotted lines corresponding to the histogram color with averages printed in the legend. (**B**) FRET efficiency for 1 and 2 $\mu$M mixtures based on the lifetime distributions in (**A**), calculated according to equation (7).

The mean lifetime of 2 $\mu$M Fluorescein in PBS measured 3.91 ns using a single-exponential decay model (Fig. 5A). 1 or 2 $\mu$M mixtures of Fluorescein and TAMRA had a mean lifetime decay of 3.88 ns. This small reduction in the lifetime of Fluorescein amounted to a mean FRET efficiency of 1 $\pm \mathrm{2 \% }$ according to Equation (7).

7 $$\begin{array}{l}E=1-\frac{{\tau }_{DA}}{{\tau }_{D}}\end{array}$$

**Figure 6:**
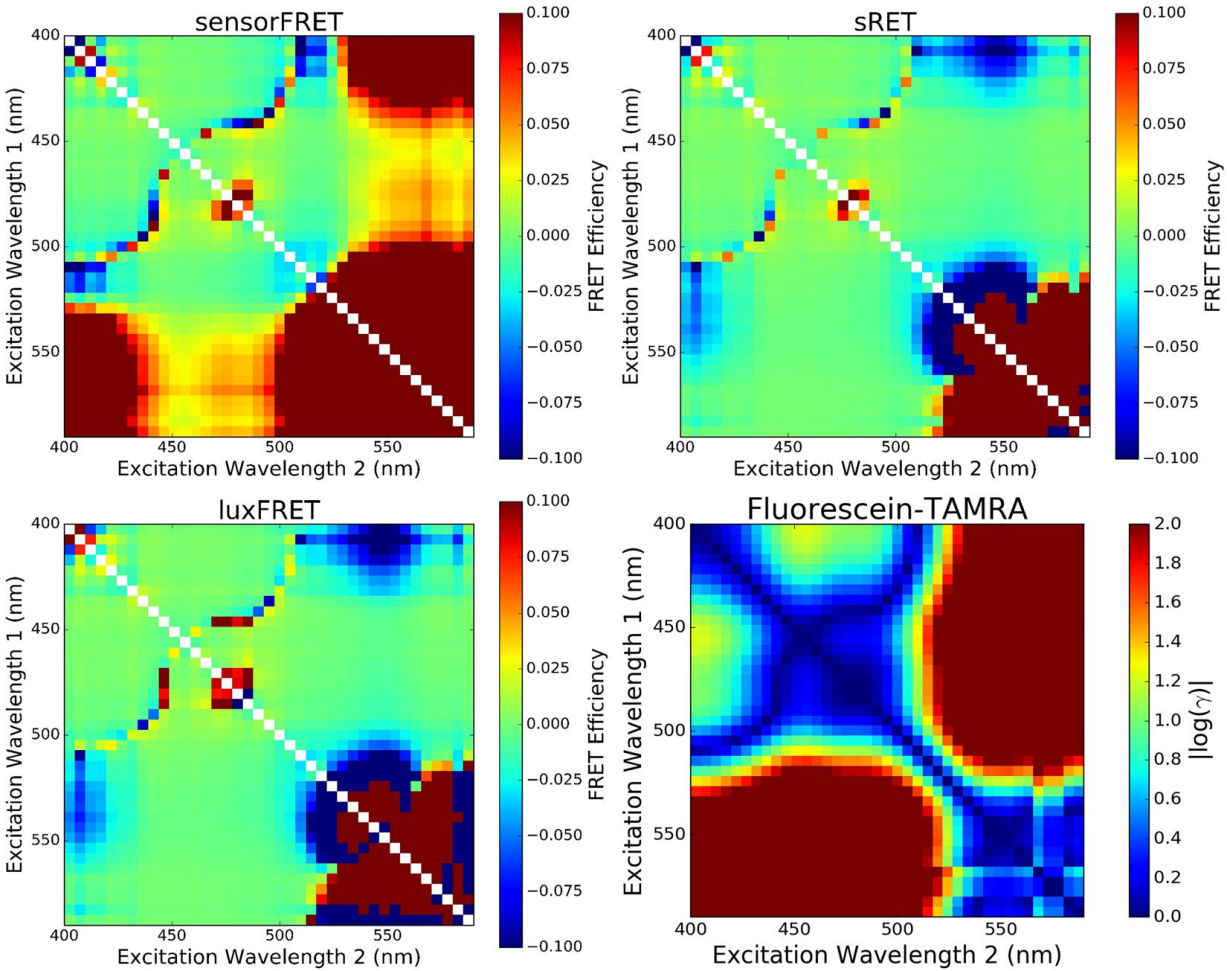
Excitation Pairing Matrices: Excitation-emission recordings shown in Fig. 4F were used to generate pairs of emission spectra at two different excitation frequencies: ${F}_{1}({\lambda }_{e{x}_{1}},{\lambda }_{em})$,${F}_{2}({\lambda }_{e{x}_{2}},{\lambda }_{em})$, where *F*_1_ is the spectra at excitation frequency 1, ${\lambda }_{e{x}_{1}}$ the fixed excitation frequency 1, and ${\lambda }_{em}$ the entire emission spectrum. Each pair of spectra were used as inputs into the spectral unmixing algorithms (LuxFRET, sRET, and SensorFRET). Every possible paired input spectra were used to output an estimated FRET efficiency that is color coded in each matrix. Ideally, all pixels would register 0% FRET (green), however certain excitation pairs have either poor signal to noise ratios or $\gamma \approx 1$. sRET and LuxFRET showed identical error sensitivity to various input frequencies and SensorFRET showed slightly less error tolerance. All algorithms fail at the red-edge of the spectrum where donor signal was absent or where gamma is near unity.

Measurements of the complete excitation and emission profile of Fluorescein and TAMRA combined with fluorescent lifetime imaging confirmed the dye pair do not transfer energy at 2 $\mu$M concentration. Next, the Fluorescein/TAMRA mixture was used as a negative FRET control to quantitatively compare the performance of SensorFRET with sRET and LuxFRET. Using spectra from the EEM of the dye mixture (Fig. 4F), FRET efficiencies were estimated for all possible excitation frequency pairings with a sampling resolution of 5 nm (Fig. 6). These paired excitation matrices reveal that all spectral FRET algorithms fail at the red-end of the spectrum where the donor magnitude is undetectable above noise at both excitation frequencies. sRET and luxFRET are able to accurately determine the FRET efficiency as long as the donor signal is measurable at one of the two frequencies, whereas SensorFRET requries the donor to be measurable at both frequencies. Unexpectedly, all algorithms failed at excitation frequencies with large signal to noise ratios in a symmetric pattern with respect to the identity diagonal (Fig. 6). These regions of systematic error correspond to excitation frequencies where the magnitude of the sensorFRET *γ* parameter is near 1 (Fig. 6D, $|log(\gamma )|=\mathrm{0)}$. As *γ* approaches unity, the normalized shape of the paired spectra are identical, leading to a failed FRET efficiency estimation in all the spectral FRET algorithms considered. By using the literature excitation spectra, the *γ* term can be determined for every possible excitation pairing (as shown in Fig. 6D and Supplemental Figure S5) and appropriate laser lines can be picked to avoid problematic pairings. For fluorophores without well documented excitation spectra, following the sensorFRET calibration procedure detailed in the supplemental section, any paired frequencies with *γ* near unity can be experimentally determined (Fig. 6D) and avoided as necessary. It should be noted however, that knowlege of *γ* alone is not sufficient to determine which frequency pairings will provide the best measurement precision for a given fluorophore pair. As *γ* approaches 0 or $\infty$ the difference in spectra shape is maximized and should therefore provide the most accurate bleed-through correction. In practice, other factors such as fluorophore brightness, autofluorescence, photobleaching/toxicity, and available laser lines will also influence which frequencies are most appropriate.

### Validation Using Protein FRET Standards

In order to experimentally verify the SensorFRET approach using a positive FRET control, 3T3 cell cultures were transfected with plasmid DNA encoded to produce soluble Cerulean-Venus FRET constructs with a known FRET efficiency. Analysis of the C32V FRET standard is shown in detail in this section, while results for other FRET standards (CTV, C5V, and VCV) are provided in Table 1. Spectral images of the same cells were acquired using both the 405 and 458 nm wavelength laser lines for single photon microscopy. Two photon excitation at 850 and 920 nm was also used to characterize the CTV, C32V, and C5V FRET standards provided in Table 1. These excitation pairs provided both high signal to noise and *γ* terms which are not close to 1 (see Supplementary Figure S5). Both single and two photon imaging modalities yielded FRET efficiency values in line with those reported in literature, including the VCV construct where the $\frac{acceptor}{donor}$ ratio is not 1:1^20,28^.

**Table 1:** Comparison of SensorFRET measurements (using both single and two photon excitation) to sRET and FLIM-FRET measurements for established FRET standards.

Method	FRET Efficiency			
FRET Standard	CTV	C32V	C5V	VCV
SensorFRET(405/458 nm)	2.1 ± 5.6 (n = 14)	36.2 ± 2.9 (n = 23)	43.8 ± 6.5 (n = 24)	70.7 ± 3.1 (n = 62)
SensorFRET(850/920 nm)	0.8 ± 5.3 (n = 16)	30.7 ± 5.4 (n = 10)	47.5 ± 3.8 (n = 12)	NA
sRET	1.7 ± 7.0 (n = 12)^*a*^	29.5 ± 8.0 (n = 60)b^*b*^	41.5 ± 8.7 (n = 60)^*b*^	69.7 ± 5.7 (n = 6)^*a*^
FLIM-FRET	6.3 ± 2.6 (n = 30)^*a*^	33.3 ± 4.5 (n = 10)^*b*^	44.3 ± 1.7 (n = 10)^*b*^	64.5 ± 3.2 (n = 20)^*a*^

Values are reported as mean  ±  std (n = # of cells measured). Values denoted with ^*a*^were obtained from Thaler *et al*.^20^. Values denoted with ^*b*^were obtained from Koushick *et al*.^28^.

**Figure 7:**
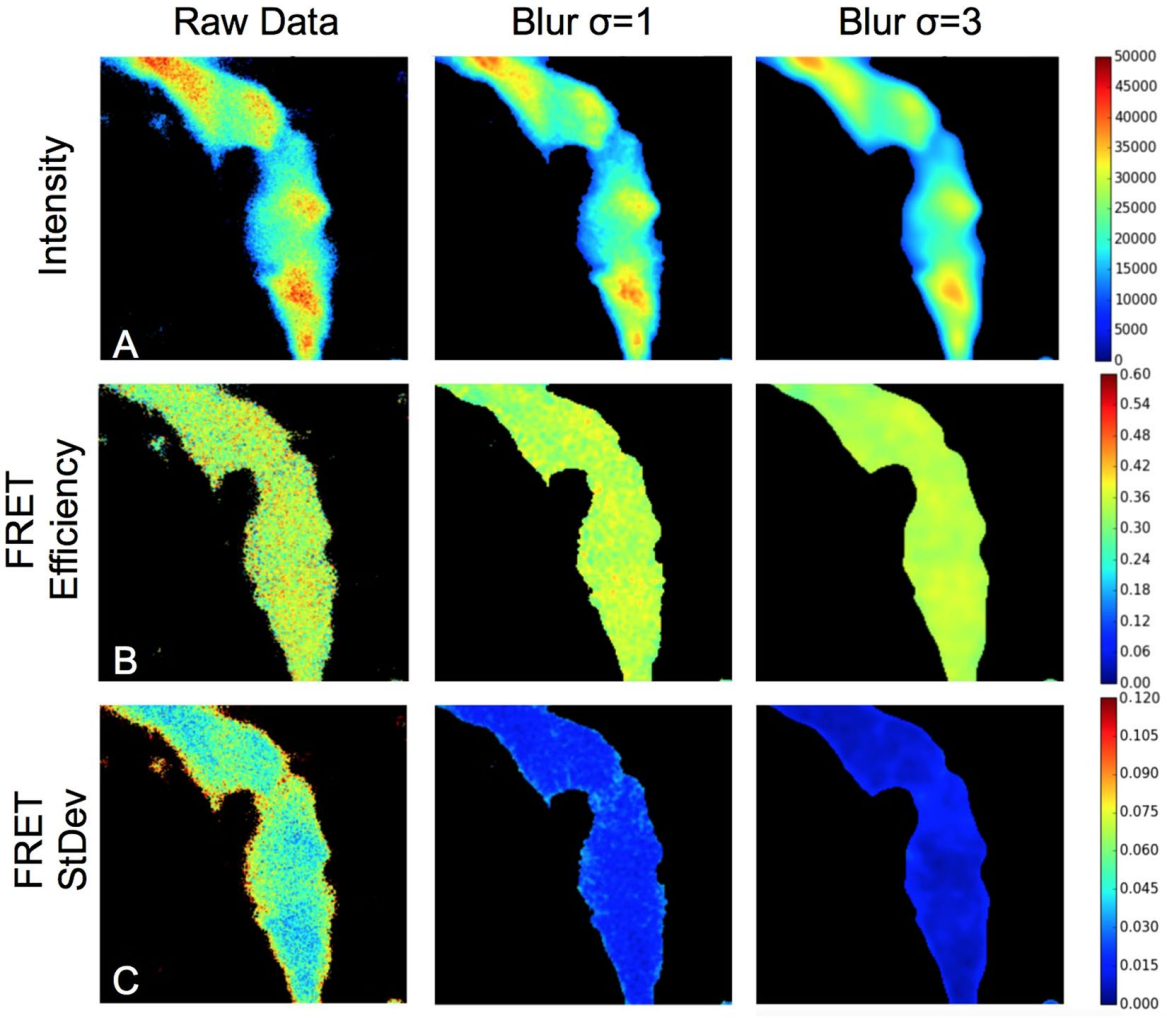
Colormap images of cells expressing the C32V FRET standard excited at 458 nm with different levels of Gaussian image blurring. Row (**A**) shows the peak intensity of the spectra for each pixel (irrespective of wavelength). Row (**B**) shows the calculated FRET efficiency for each pixel. Row (**C**) shows the expected standard deviation of the FRET efficiency for each pixel based on the residual.

In addition to the raw images, a Gaussian blur filter with $\sigma$ = 1 and 3 pixels was used to generate images with reduced spectra noise (in exchange for reduced spatial resolution), as shown in Fig. 7A. Using each of the spatially aligned images and the *γ* term determined from the literature (*γ* = 0.045), the FRET efficiency is calculated on a per-pixel basis as shown in Fig. 7B. Finally, the normalized fit residual calculated for each pixel is used to determine the expected error in the FRET efficiency on a per pixel basis (Fig. 7C), based on the simulated standard deviation vs residual curve shown in Fig. 3C. Notice that the measured FRET efficiency is uncorrelated with the measured intensity, which indicates that there is no appreciable intermolecular FRET contribution (Fig. 7A,B and Supplementary Figure S6). The predicted standard deviation is inversely correlated with the spectra intensity which is expected because the signal to noise ratio of the spectra increases as the intensity increases, due to the Poissonian nature of the noise. Any acquisition parameter change which improves the signal to noise (eg. increased laser power, decreased scan speed, lower resolution camera binning) should also have the effect of reducing the FRET standard deviation. Blurring was used simply as a convenient method of improving the spectral signal to noise on otherwise identical datasets.

In their characterization of these same C32V standards, Koushik *et al*.^28^ reported a FRET efficiency range of 29.5 ± 8.0 using sRET, and 33.3 ± 4.5 using FLIM-FRET (mean ± std of 60 and 10 cells respectively). The measured FRET efficiency of the pixels in Fig. 7B were 33.3 ± 14.2, 34.1 ± 2.7, and 34.2 ± 1.6 (mean ± std of individual pixels) for the raw, $\sigma =1$, and $\sigma =3$ images, respectively, which fall within the range of reported FRET efficiency.

**Figure 8:**
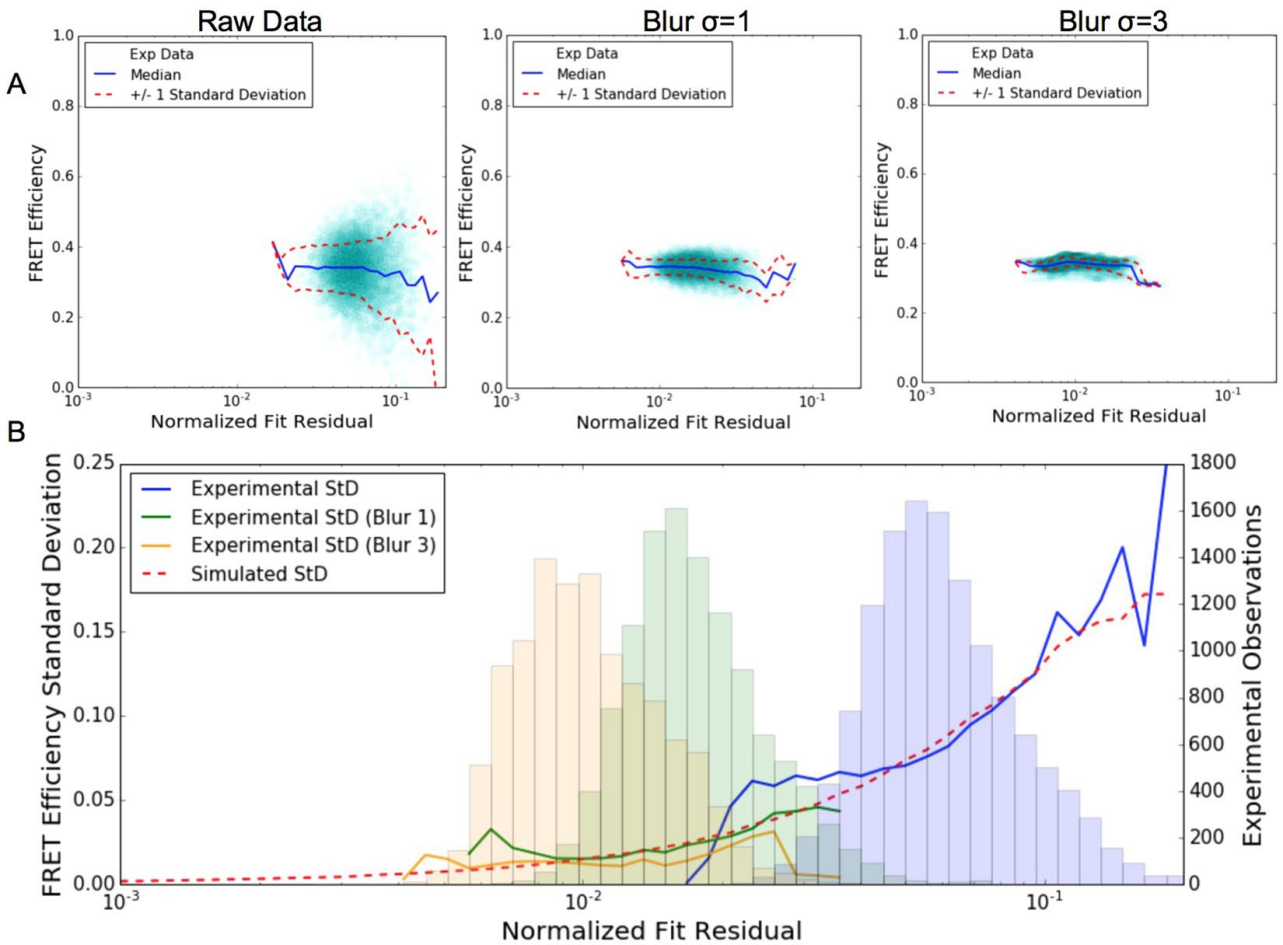
Comparison of experimental efficiency error to simulated behavior. (**A**) Shows FRET efficiency vs normalized fit residual for all of the pixels in each of the images in Fig. 7. (**B**) Comparison of experimental and simulated standard deviations showing strong agreement between the two. The experimental standard deviation estimates are only valid where there is a significant number of pixels to estimate it with so corresponding histograms are provided on the secondary axis.

Since the C32V FRET construct should have a spatially uniform efficiency, we are able to aggregate pixels to determine the standard deviation as a function of the normalized fit residual as shown in Fig. 8A. There is strong agreement with the simulated standard deviation where there are a large number of pixels at that residual level to estimate the experimental standard deviation (Fig. 8B). The curves diverge at the tails of the pixel histograms where there are much fewer pixels to calculate the standard deviation.

### Application of SensorFRET and Noise Model: Measuring Force on E-cadherin using a FRET-force Probe

Although biosensors based on unimolecular FRET constructs have a wide range of applications, they have been shown to be particularly useful in understanding how cellular forces affect biological processes^12,14,31–33^. In this section we show how force maps can be generated from spectral imaging of MDCK cells expressing force sensitive unimolecular Teal-Venus FRET constructs (TV40)^12^.

To determine the FRET efficiency in both the loaded and unloaded conditions, two cell lines were developed with TV40 FRET sensors integrated into E-cadherin^33^. The first of these, denoted TL for tailless, generates TV40 labeled E-cadherin proteins which cannot attach to the rest of the cytoskeleton, preventing any stress from being applied to the sensor. The average FRET efficiency determined from these cells gives a measure of the FRET efficiency of the sensor in the unloaded condition. The second of these, denoted TS for tension sensor, function similarly to endogenous E-cadherin but transfers load through the FRET construct, allowing any decrease in FRET efficiency relative to the TL sample to be interpreted as increased force on the E-cadherin proteins. Since the linker protein that separates Teal and Venus in TV40 behaves as an elastic spring^13^, decreases in FRET efficiency in the TS sample can be transformed to changes in force on E-cadherin if we assume the change in FRET efficiency is a result of changing distance between Teal and Venus.

**Figure 9:**
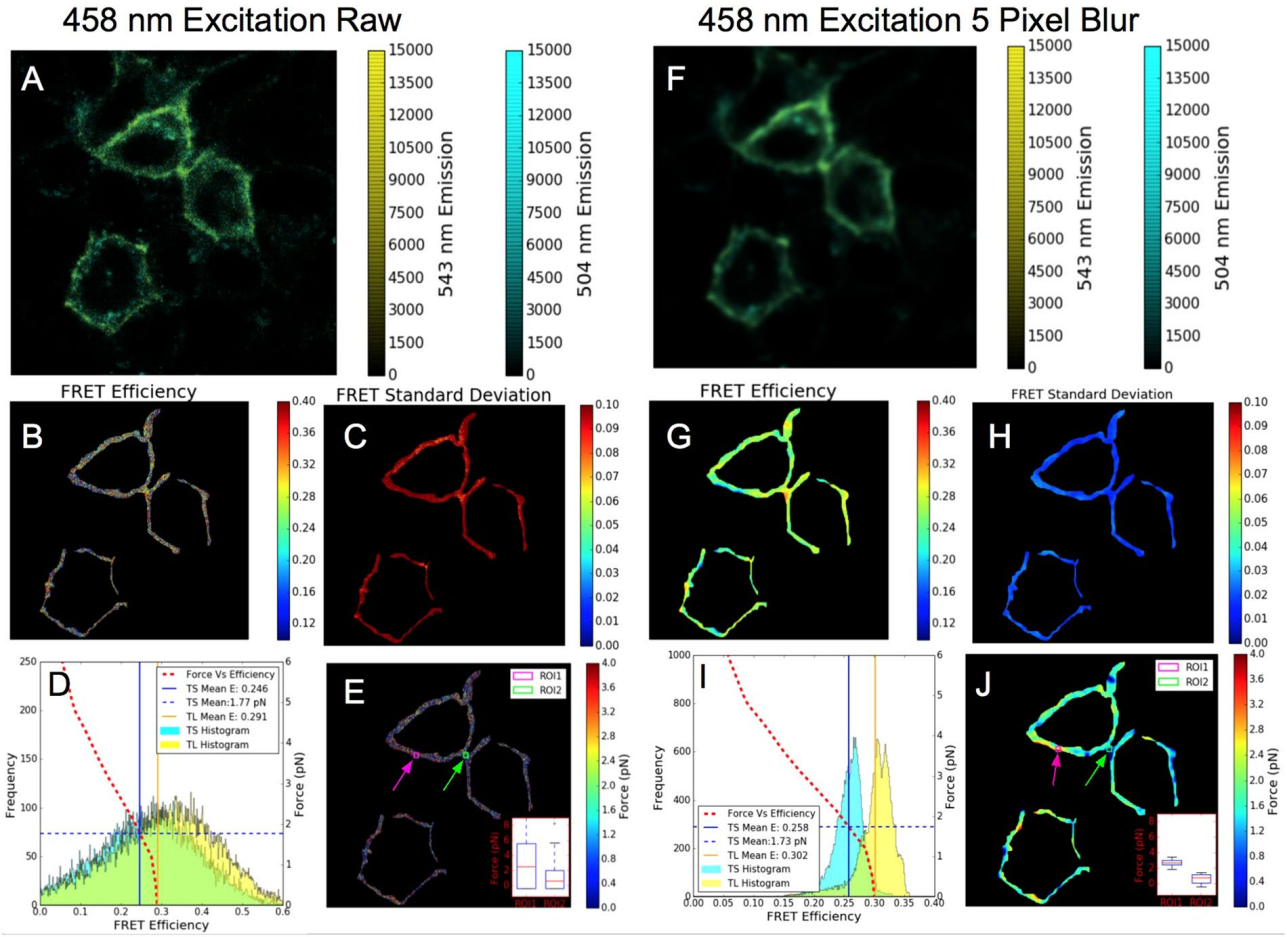
SensorFRET analysis of MDCK cells expressing TV40 unimolecular FRET constructs incorporated into E-cadherin transmembrane proteins (denoted TS). (**A**–**E**) show analysis for the raw data while (**F**–**J**) show analysis results for the same data after a 5 pixel Gaussian blur. Panels A/F shows a false color intensity image (by overlaying the Teal and Venus peak intensities), panels B/G show the FRET efficiency maps calculated on a per pixel basis, panels C/H show the FRET efficiency standard deviation (estimated from Supplemental Figure S7, using the measured FRET efficiency and Normalized Fit Residual). Panels D/I show the histogram of measured FRET efficiency values taken from B/G on the primary axis along with the unloaded TL control (image not shown), while the secondary axis shows the estimated Force (in pN) as a function of FRET efficiency. The intersection of the median FRET Efficiency for the tension sensor and the FRET vs Force plot implies the median force per molecule is approximately 1.7 pN. Panels E/J show the estimated force mapped on a per pixel basis, with the inset boxplots showing that differences can only be discerned between regions within a single image once the image is blurred.

As with the Cerulean-Venus FRET standards, spectral images were acquired at 458 and 405 nm excitation wavelengths. These images, in conjunction with the *γ* term determined from literature^34^ ($\gamma =0.101$ for the Teal-Venus/405–458 nm fluorophore/excitation combination, see Supplementary Figure S5), enables the calculation of FRET efficiency on a pixel by pixel basis as shown in Fig. 9B,G. Since the FRET efficiency is not spatially uniform (in contrast to the Cerulean Venus FRET standards), in order to determine the expected standard deviation, residual vs standard deviation curves (analogous to Fig. 3C) were simulated over a range of FRET efficiencies, as shown in Supplementary Figure S7. Interpolation of these curves allows the expected standard deviation to be determined for any given pixel as a function of the measured FRET efficiency and normalized fit residual, as shown in Fig. 9C,H. It is clear from these plots that the blurring procedure does not affect the measured FRET efficiency on average, but significantly reduces the standard deviation of the individual pixel measurements. One of the main advantages of this approach is that it allows the user to quantify their measurement error and reduce it using blurring (or other filtering methods) until it reaches a level which is acceptable for their particular experimental requirements at a cost of reduced spatial resolution. In this particular application, we aimed to reduce the FRET pixel standard deviation below 0.02 (arbitrarily chosen to allow differences in the TS and TL histograms to be readily observed). The blurring was increased until this condition was met, finally requiring a 5 pixel gaussian blur as shown in Fig. 9F–J. This noise characterization is particularly useful for determining whether the pixel to pixel variance in FRET is due to measurement uncertainty derived from the instrumentation or whether the variance results from real changes in the distance or orientation of the donor-acceptor pair. Since the standard deviation inferred from the normalized fit residual only captures the variance caused by the instrumentation, we can infer that additional measurement variance above the predicted level (Supplementary Figure S7) likely results from physical changes (orientation, distance, or environment) to the fluorophores in the biosensor.

In order to convert the measured FRET efficiency to force, the mechanical response of the peptide chain linking the two fluorophores must be known. For the particular peptide linker used in the TV40 FRET construct, the FRET efficiency vs load behavior was characterized in a previous study using optical tweezers to apply loads to single molecules^12,13^. Because the Cy3 and Cy5 fluorophores used by Grashoff *et al*. have a different effective fluorophore diameter and Forester radius than the Teal and Venus fluorophores, the FRET efficiency vs load response must be scaled such that the mean FRET efficiency of the TL expressing cells (E = 0.315) corresponds to 0 pN load and as the load approaches $\infty$ the FRET efficiency asymptotes towards E = 0.

This FRET efficiency vs force calibration curve is shown in Fig. 9D,I, superimposed on the histogram of the measured FRET efficiencies for the whole image. These plots show that by averaging the pixels in the whole image there is a measurable difference in the FRET efficiency between the TS and TL, corresponding to a $1.7\pm 4.8$ pN (raw) $1.7\pm 0.5$ pN (blurred) force applied to the E-cadherin on average. This force estimate is in agreement with measurements of E-cadherin-TS by Borghi *et al* ^33^. The force may also be calculated on a per pixel basis, as shown in Fig. 9E,J, for the raw and blurred data, respectively. Drawing strong conclusions about differences in force between regions within a single image is challenging when using the raw data set due to the large amount of variance, shown in the inset boxplots of Fig. 9E generated from the magenta and green regions denoted in the force map. In the blurred image, however, the variance in the same regions is much less than the difference in the mean observed (inset boxplot of Fig. 9J), showing that statistically significant differences can be observed when comparing different parts of the cell boundaries in the same image.

In this work, we demonstrate the SensorFRET analysis approach allows simultaneous measurements of spectral bleed-through and FRET efficiency on a per-pixel basis using spectral imaging microscopy. SensorFRET does not require single fluorophore references as long as the normalized excitation and emission spectra of the sensor fluorophores are known. The cell environment and cell type will affect the autofluorescence contribution to the measured spectra and any additional fluorescent labels will also contribute to the signal. As the magnitude of these contributions is variable with respect to the sensor expression, the most appropriate way to account for these effects is to measure the normalized emission spectra (using a cell culture with no labels for the autofluorescence and single label controls for any additional labels) and unmix these components along with the donor and acceptor. If the emission spectra from autofluorescence or additional labels are similar to donor or acceptor spectra, this can add uncertainty to the unmixing process and reduce the accuracy of the FRET measurement. If the cellular environment (pH,redox, etc.) leads to significant distortions in the fluorescent emission of the donor or acceptor used, a one time calibration measurement can be used to calculate *γ* experimentally and correct for any differences in the emission spectra. The validity of this approach was verified by simulation and experimental measurements using standard reference dyes (Fluorescein and TAMRA) used as a negative FRET control and unimolecular FRET standards encoding Cerulean and Venus fluorescent proteins used as positive FRET controls. Noiseless FRET simulations demonstrated the mathematical basis of SensorFRET while simulations with Poisson (ie. shot) and thermal noise showed the accuracy and precision of SensorFRET was indistinguishable from luxFRET, pFRET, and sRET approaches^17,19,20^. To our knowledge, Fluorescein and TAMRA are the first pair of fluorescent dyes to be used as FRET controls and could be useful to researchers that need to verify the implementation of their FRET analysis. In theory, any FRET pair and excitation wavelengths could be used with this method, however, excitation wavelengths must be chosen such that sufficient donor fluorophore brightness can be achieved at both wavelengths to improve signal to noise. Competing with this requirement, however, is the limitation that as *γ* approaches 1, any error in the *γ* estimate will have a larger effect on the calculation of the acceptor direct excitation and lead to significant errors in the measured FRET efficiency. For the FRET sensors used in this study (Cerulean-Venus and Teal-Venus) *γ* was close to 0 (CV = 0.045 and TV = 0.101) and the excitation frequencies (405 and 458 nm) were sufficient to excite a large proportion of donor molecules at either frequency. We also demonstrate how this FRET method and noise model can be used to measure piconewton scale forces on the force bearing cell junction molecule E-cadherin in MDCK epithelial cells. The data show that E-cadherin molecules in MDCK cells have a median resting force of 1.7 pN + − 4.8 pN. Applying a 5-pixel radius gaussian blur reduced the standard deviation to 0.5 pN, enabling statistically significant differences to be spatially resolved in a single image. It is important to note pixel-based FRET efficiency error images shown in Fig. 9C,H rely on simulations that used experimentally determined emission shapes and gamma. Estimating pixel-wise FRET efficiency error by use of literature spectra may bias these estimates if the spectral detector is improperly calibrated or gamma is shifted in the cellular environment.

By greatly simplifying the experimental requirements for quantitative FRET determination, the SensorFRET approach allows this nano characterization technique to be accessible to a much broader range of the research community.

## Methods

### Simulations

Simulations of FRET spectra were created using the ipython notebook. Idealized FRET spectra were simulated using parameters available in the literature. The FRET spectra, ${F}_{DA}$, is a function of the emission wavelength, ${\lambda }_{em}$, excitation wavelength, ${\lambda }_{ex}$, and FRET efficiency, *E*, was calculated according to:

8 $$\begin{array}{c}{F}_{DA}({\lambda }_{em},{\lambda }_{ex},E)=I({\lambda }_{ex})\ast [DA]\ast [{s}_{D}\ast {\hat{\varepsilon }}_{D}({\lambda }_{ex}\mathrm{)((1}-E)\ast {Q}_{D}\ast {\hat{e}}_{D}({\lambda }_{em})\\ \quad \quad \quad \quad \quad \quad +E\ast {Q}_{A}\ast {\hat{e}}_{A}({\lambda }_{em}))+{s}_{A}\ast {\hat{\varepsilon }}_{A}({\lambda }_{ex})\ast {Q}_{A}\ast {\hat{e}}_{A}({\lambda }_{em})]\end{array}$$

$$\begin{array}{ccc}\quad \quad \quad \quad \quad I({\lambda }_{ex}) & = & {\rm{intensity}}\,{\rm{at}}\,{\rm{the}}\,{\rm{sample}}\\ \quad \quad \quad \quad \quad [DA] & = & {\rm{concentration}}\,{\rm{of}}\,{\rm{FRET}}\,{\rm{construct}}\\ {\hat{\varepsilon }}_{D}({\lambda }_{ex})\,{\rm{and}}\,{\hat{\varepsilon }}_{A}({\lambda }_{ex}) & = & {\rm{normalized}}\,{\rm{don}}\,{\rm{or}}\,{\rm{and}}\,{\rm{acceptor}}\,{\rm{excitation}}\,{\rm{spectra}}\\ {\hat{e}}_{D}({\lambda }_{em})\,{\rm{and}}\,{\hat{e}}_{A}({\lambda }_{em}) & = & {\rm{normalized}}\,{\rm{donor}}\,{\rm{and}}\,{\rm{acceptor}}\,{\rm{emission}}\,{\rm{spectra}}\\ \quad \quad \quad \quad {s}_{D}\,{\rm{and}}\,{s}_{A} & = & {\rm{scaling}}\,{\rm{factors}}\,{\rm{for}}\,{\rm{the}}\,{\rm{donor}}\,{\rm{and}}\,{\rm{acceptor}}\,{\rm{excitation}}\,{\rm{spectra}}\\ \quad \quad \quad \,\,{Q}_{D}\,{\rm{and}}\,{Q}_{A} & = & {\rm{quantum}}\,{\rm{efficiencies}}\,{\rm{of}}\,{\rm{the}}\,{\rm{donor}}\,{\rm{and}}\,{\rm{acceptor}}\end{array}$$

The normalized emission spectra, excitation spectra and quantum efficiencies were all readily available in the literature for both 1 photon^23^ and 2 photon^20^ excitation. In order to have comparable spectral resolution to the experimental results, the literature emission spectra were re-sampled at 32 wavelengths between 416 and 718 nm. Values for the intensity, concentration and excitation spectra at 405 and 458 were chosen such that the spectral shape at a given FRET efficiency matches what is observed experimentally (ie. $[I({\lambda }_{405})\ast [DA]\ast {s}_{D}\ast {\hat{\varepsilon }}_{D}$ $({\lambda }_{405})]=242280,[I({\lambda }_{458})\ast [DA]\ast {s}_{D}\ast {\hat{\varepsilon }}_{D}({\lambda }_{458})]=32334$, $[I({\lambda }_{405})\ast [DA]\ast {s}_{A}\ast$ ${\hat{\varepsilon }}_{A}({\lambda }_{405})]=5802$, and $[I({\lambda }_{458})\ast [DA]\ast {s}_{A}\ast {\hat{\varepsilon }}_{A}({\lambda }_{458})]=12793$).

For the luxFRET, sRET, and pFRET methods it was also necessary to simulate single fluorophore spectra for the calibration processes required by each of these analysis approaches using the following equation.

9 $${F}_{X}({\lambda }_{em},{\lambda }_{ex})=I({\lambda }_{ex})\ast [X]\ast {s}_{X}\ast {\hat{\varepsilon }}_{X}({\lambda }_{ex})\ast {Q}_{X}\ast {\hat{e}}_{X}({\lambda }_{em})$$

where X denotes either D or A for the donor or acceptor single fluorophore respectively. The $I({\lambda }_{ex})$ term was maintained between the FRET construct spectra and calibration spectra and the [*X*] terms were maintained at both excitation frequencies, as required by the luxFRET, sRET, and pFRET analysis approaches. Excitation wavelengths of 405 and 458 were used in the simulation of SensorFRET, luxFRET, and sRET, while excitation wavelengths of 458 and 515 were simulated for pFRET. The simulated noiseless spectra are shown in Supplementary Figure S2.

### Cell culture

NIH-3T3 mouse fibroblasts and Madin-Darby Canine Kidney Epithelial Cells (MDCK) were cultured in Dulbecco’s Modified Eagle Medium (DMEM) with 10% fetal calf serum and 500ug/mL of Penniciln-Strepomycin antibiotics. Cells were maintained in an atmospherically controlled incubator at 37 degrees celsius and 5% CO2 atmosphere. Media were changed every other day.

### DNA Preparation

Soluble Cerulean-Venus FRET standards were gifts from Steven Vogel^28^ (addgene numbers: 26394-C5V,26395-C17V,C32V-26396,27803-CTV,27799-C5A,VCV-27788) and received from addgene depository. Venus plasmid was a gift from Michael Davidson (Venus-54859) received from addgene depository. E-cadherin tension sensors (denoted TS and TL) were gifts of Alex Dunn^33^. Plasmids were received as E-coli vectors expressing C5V, C32V, CTV, CTA, and Venus plasmids. E-coli cultures were amplified in LB-broth overnight and plasmid DNA was isolated by afinity column purification using the NucleoBond® Xtra Midi kit distributed by Macherey-Nagel per manufacturer instructions.

### Plasmid Transfection

DNA plasmids were transfected into cells using Lipofectamine 2000 or 3000 (Life Technologies) per manufacturer instructions. In all experiments cells were allowed to adhere to fibronectin coated glass bottom dishes or coverslips overnight before imaging.

### Single-Photon Imaging

Images were acquired from cells grown on glass bottom dishes on an inverted Zeiss LSM 710 confocal using a either 405 nm or 458 nm excitation wavelengths from an argon laser source. A plan-apochromat 20x objective lens (NA = 0.8) was used for all images involving the FRET standards or single fluorophore reference samples. Live cells expressing either soluble Cerulean-5-Amber or Venus were imaged in spectral mode using a 32-channel spectral META detector to record spectral fingerprints of Cerulean and Venus fluorophores respectively. For the FRET standards, images were captured in spectral mode with the emission frequencies spanning 460–720 nm with 10 nm spacing per channel. Images were captured in 16 bit mode, scanned bi-directionally, and averaged 4 times.

### Two-Photon Imaging

Images were acquired from cells grown on glass bottom dishes on an upright Zeiss LSM 510 META NLO multi-photon laser scanning microscope with water immersion objectives. Images were captured with an internal de-scanned meta detector with emission frequencies spanning 367–699 nm at 11 nm spacing between channels. Excitation frequencies (850 nm and 920 nm) were tuned using a Spectra-Physics Mia-Tai broadband tunable Ti:sapphire laser.

*Fluorometer Measurements* Fluorescein and carboxy-tetramethylrhodamine (TAMRA) were purchased from Sigma in their Reference Dye Sampler Kit (R14782). Stock 1 mM solutions were diluted in phosphate buffered saline (PBS, pH 7.4) to final concentrations (1uM). Working solutions were transferred to a 3.5 mL 4-sided Quartz cuvette with a path length of 10mm and an optical working range of 334 to 2500 nm (Starna 3-G-10). All excitation and emission measurements were recorded by a Varian Cary Eclipse Flourometer (SN:EL00043440) in 3D mode. Excitation and emission wavelengths were captured at 5 and 2 nm increments respectively. Slit widths for the excitation and emission were set to 5 and 2.5 nm respectively. The excitation and emission bands spanned from (400–700 nm) and (475–750 nm) respectively. All measurements were averaged for 0.1 s and the PMT voltage was adjusted such that the peak emission intensity approached approximately 80% of the saturation intensity of the detector. All data analysis was performed in the ipython notebook where the mixed spectra were deconvolved using the non-negative least squares (nnls) scientific python package.

### FLIM Imaging

Fluorescent lifetime imaging was performed on a two-photon Zeiss 780 NLO microscope equipped with a 32-channel descan spectral GaAsP (Gallium Arsenide Phosphide) detector. The Zeiss 780 was coupled with a Ti:Sapphire laser (Chameleon Vision-II, ultrafast) tunable from 680 nm to 1080 nm. The excitation frequency was tuned 860 nm. To capture photons from fluorescein-only an HQ510–50m dichroic filter was used. A Becker and Hickl FLIM hybrid detector (HPM-100–40) coupled to the 780 NDD port was used for time-correlated single photon counting (TCSPC). To ensure instrument calibration, the standard dye fluorescein was used at a previously published concentration of 50 $\mu$M^28^. Specifically, stock 1 mM flourescein pre-dissolved in DMSO was diluted in sodium borate buffer (pH 9) to a final concentration of 50uM. THe 50 $\mu$M diluted fluorescein was pipetted onto a glass slide and the fluorescent droplet was immediately imaged at 22 C. Photons were captured for total duration of 30 seconds at rate of approximately 200,000 events per second. Lifetime images were imported into Becker and Hickl software SPCI and fit using a single exponential model. The fitting shift had to be manually fixed to value that minimized the Chi-square statistic. The offset was manually fixed to 0.

### Data Availability

Simulated Cerulean-Venus spectra for benchmarking FRET analysis approaches are available at 10.6084/m9.figshare.5573542.v1. Other datasets generated during and/or analyzed during the study are available from the corresponding author on reasonable request.

## Electronic supplementary material

Supplementary information
