# Supplementary material for: SensorFRET: A Standardless Approach to Measuring Pixel-based Spectral Bleed-through and FRET Efficiency using Spectral Imaging
Source: Sci Rep. 2017 Nov 15;7:15609. doi: 10.1038/s41598-017-15411-8 (PMC5688180; doi:10.1038/s41598-017-15411-8)
Supplement: Supplementary file 1 — Supplementary information [file 41598_2017_15411_MOESM1_ESM.pdf]

SensorFRET: A Standardless Approach to Measuring Pixel-based Spectral Bleed-through and FRET Efficiency using Spectral Imaging

Paul Arsenovic, Carl Mayer, and Daniel Conway

Supplementary Note 1: Derivation of the SensorFRET method

The magnitude and shape of a single donor fluorophore's spectra can be described as:

$$F_D(\lambda_{em},\lambda_{ex}) = I(\lambda_{ex}) * [D] * s_D * \hat{\varepsilon}_D(\lambda_{ex}) * Q_D * \hat{e}_D(\lambda_{em}) \tag{S1}$$

$F_D(\lambda_{em},\lambda_{ex})$ = complete fluorescent spectra
λ_{em} = emission wavelengths
λ_{ex} = excitation wavelengths
$I(\lambda_{ex})$ = laser excitation power
[D] = concentration of donor molecules
s_D = donor excitation coefficient scalar
$\hat{\varepsilon}_D(\lambda_{ex})$ = normalized excitation spectrum
Q_D = quantum efficiency
$\hat{e}_D(\lambda_{em})$ = normalized emission spectrum

The magnitude and shape of a single FRET sensor's spectra can be described as:

$$F_{DA}(\lambda_{em},\lambda_{ex}) = I(\lambda_{ex}) * [DA]\Big[s_D * \hat{\varepsilon}_D(\lambda_{ex})\big((1-E) * Q_D * \hat{e}_D(\lambda_{em}) + E * Q_A * \hat{e}_A(\lambda_{em})\big) + s_A * \hat{\varepsilon}_A(\lambda_{ex}) * Q_A * \hat{e}_A(\lambda_{em})\Big] \tag{S2}$$

$F_{DA}(\lambda_{em},\lambda_{ex})$ = complete fluorescent FRET spectra
E = FRET efficiency
$\hat{\varepsilon}_A(\lambda_{ex})$ = normalized acceptor excitation spectrum
$\hat{e}_A(\lambda_{em})$ = normalized acceptor emission spectrum
s_A = acceptor excitation scalar
Q_A = quantum efficiency of the acceptor
[DA] = concentration of FRET molecules

The total measured fluorescent signal can be attributed to both paired FRET sensor molecules and unpaired fluorophores. Even in unimolecular FRET constructs, signal from unpaired fluorophores can arise from selective bleaching, quenching, or improper protein folding. The total signal can be thought of as the sum of five components, the FRET Donor signal, the FRET Acceptor signal, the FRET Acceptor Direct Excitation, the Unpaired Donor, and Unpaired Acceptor defined here as:

$$F_{DA}(\lambda_{em},\lambda_{ex}) = \text{FRET Donor} + \text{FRET Acceptor} + \text{FRET Acceptor Direct Excitation} + \text{Unpaired Donor} + \text{Unpaired Acceptor} \tag{S3}$$

$$\text{FRET Donor} = I(\lambda_{ex}) * [DA] * s_D * \hat{\varepsilon}_D(\lambda_{ex}) * (1-E) * Q_D * \hat{e}_D(\lambda_{em})$$
$$\text{FRET Acceptor} = I(\lambda_{ex}) * [DA] * s_D * \hat{\varepsilon}_D(\lambda_{ex}) * E * Q_A * \hat{e}_A(\lambda_{em})$$
$$\text{FRET Acceptor Direct Excitation} = I(\lambda_{ex}) * [DA] * s_A * \hat{\varepsilon}_A(\lambda_{ex}) * Q_A * \hat{e}_A(\lambda_{em})$$
$$\text{Unpaired Donor} = I(\lambda_{ex}) * [D] * s_D * \hat{\varepsilon}_D(\lambda_{ex}) * Q_D * \hat{e}_D(\lambda_{em})$$
$$\text{Unpaired Acceptor} = I(\lambda_{ex}) * [A] * s_A * \hat{\varepsilon}_A(\lambda_{ex}) * Q_A * \hat{e}_A(\lambda_{em})$$

Linear unmixing using the normalized emission shapes of the donor and acceptor fluorophores ($\hat{e}_D$and $\hat{e}_A$) yields the magnitude of the donor and acceptor components:

$$\begin{aligned} F_{DA}(donor, \lambda_{exi}) =& I(\lambda_{exi}) * s_D * \hat{\varepsilon}_D(\lambda_{exi}) * Q_D\big([DA] * (1-E) + [D]\big) \\ F_{DA}(acceptor, \lambda_{exi}) =& I(\lambda_{exi}) * [DA] * s_D * \hat{\varepsilon}_D(\lambda_{exi}) * E * Q_A \\ &+ I(\lambda_{exi}) * s_A * \hat{\varepsilon}_A(\lambda_{exi}) * Q_A\big([DA] + [A]\big) \end{aligned} \tag{S4}$$

Taking the ratio of the donor component magnitudes at two different excitation wavelengths, we are left with a quantity that is proportional to the ratio of the donor excitation coefficients scaled by the power coefficients at excitation wavelengths 1 and 2 as shown below:

$$\frac{F_{DA}(donor, \lambda_{ex2})}{F_{DA}(donor, \lambda_{ex1})} = \frac{I(\lambda_{ex2}) * s_D * \hat{\varepsilon}_D(\lambda_{ex2}) * Q_D\big([DA] * (1-E) + [D]\big)}{I(\lambda_{ex1}) * s_D * \hat{\varepsilon}_D(\lambda_{ex1}) * Q_D\big([DA] * (1-E) + [D]\big)} = \frac{I(\lambda_{ex2}) * \hat{\varepsilon}_D(\lambda_{ex2})}{I(\lambda_{ex1}) * \hat{\varepsilon}_D(\lambda_{ex1})} \equiv \alpha \tag{S5}$$

We call this quantity α, which is a scalar quantity that is constant for a given FRET sensor at a fixed pair of excitation frequencies and power ratio. This α term can then be used to scale $F_{DA}(\lambda_{ex1}, \lambda_{em})$ such that the FRET Donor and FRET Acceptor terms in S3 are equivalent at both excitation wavelengths as shown below (the equivalent FRET Donor and FRET Acceptor terms are shown in large brackets):

$$\begin{aligned} \alpha * F_{DA}(\lambda_{em}, \lambda_{ex1}) = &\Big[I(\lambda_{ex2}) * [DA] * s_D * \hat{\varepsilon}_D(\lambda_{ex2})\big((1-E) * Q_D * \hat{e}_D(\lambda_{em}) + E * Q_A * \hat{e}_A(\lambda_{em})\big)\Big] + \\ & I(\lambda_{ex2}) * \frac{\hat{\varepsilon}_D(\lambda_{ex2})}{\hat{\varepsilon}_D(\lambda_{ex1})} * s_A * \hat{\varepsilon}_A(\lambda_{ex1}) * Q_A * \hat{e}_A(\lambda_{em})\big([DA] + [A]\big) \end{aligned} \tag{S6}$$

$$\begin{aligned} F_{DA}(\lambda_{em}, \lambda_{ex2}) = &\Big[I(\lambda_{ex2}) * [DA] * s_D * \hat{\varepsilon}_D(\lambda_{ex2})\big((1-E) * Q_D * \hat{e}_D(\lambda_{em}) + E * Q_A * \hat{e}_A(\lambda_{em})\big)\Big] + \\ & I(\lambda_{ex2}) * s_A * \hat{\varepsilon}_A(\lambda_{ex2}) * Q_A * \hat{e}_A(\lambda_{em})\big([DA] + [A]\big) \end{aligned} \tag{S7}$$

Taking the difference between $F_{DA}(\lambda_{em}, \lambda_{ex2})$ and $\alpha * F_{DA}(\lambda_{em}, \lambda_{ex1})$, we are able to obtain a term related to the magnitude of the acceptor direct excitation, defined as β:

$$\begin{aligned} \beta * \hat{e}_A(\lambda_{em}) \equiv & F_{DA}(\lambda_{em}, \lambda_{ex2}) - \alpha * F_{DA}(\lambda_{em}, \lambda_{ex1}) \\ =& I(\lambda_{ex2}) * s_A * \hat{\varepsilon}_A(\lambda_{ex2}) * Q_A * \hat{e}_A(\lambda_{em})\big([DA] + [A]\big) \\ & - I(\lambda_{ex2}) * * \frac{\hat{\varepsilon}_{D2}}{\hat{\varepsilon}_{D1}} * s_A * \hat{\varepsilon}_A(\lambda_{ex1}) * Q_A * \hat{e}_A(\lambda_{em})\big([DA] + [A]\big) \\ =& I(\lambda_{ex2}) * s_A * \hat{\varepsilon}_A(\lambda_{ex2}) * Q_A * \hat{e}_A(\lambda_{em})\big([DA] + [A]\big)\left(1 - \frac{\hat{\varepsilon}_{A1}}{\hat{\varepsilon}_{A2}} * \frac{\hat{\varepsilon}_{D2}}{\hat{\varepsilon}_{D1}}\right) \\ & or \\ \beta =& F_{DA}(acceptor, \lambda_{ex2}) - \alpha * F_{DA}(acceptor, \lambda_{ex1}) \end{aligned} \tag{S8}$$

Eq. S8 shows that the scalar quantity β can be computed by least squares fitting of $\hat{e}_A$. Since $F_{DA}(\lambda_{em}, \lambda_{ex2}) - \alpha * F_{DA}(\lambda_{em}, \lambda_{ex1})$ is ideally the same shape as the acceptor emission, this approach provides a useful visual check that the direct excitation can be characterized above any noise in the signal. Alternatively, β can be calculated directly from the acceptor component magnitudes $F_{DA}(acceptor, \lambda_{ex2}) - \alpha * F_{DA}(acceptor, \lambda_{ex1})$, which is computationally less intensive than a second fitting operation.

The final parameter required to determine the acceptor direct excitation term is γ, defined as:

$$\gamma \equiv \frac{\hat{\varepsilon}_D(\lambda_{ex2}) * \hat{\varepsilon}_A(\lambda_{ex1})}{\hat{\varepsilon}_D(\lambda_{ex1}) * \hat{\varepsilon}_A(\lambda_{ex2})} \tag{S9}$$

Since the excitation spectra of most fluorophores have been previously characterized in the literature, each of the $\hat{\varepsilon}_D(\lambda_{ex2})$, $\hat{\varepsilon}_D(\lambda_{ex1})$, $\hat{\varepsilon}_A(\lambda_{ex1})$, and$\hat{\varepsilon}_A(\lambda_{ex2})$ terms can be determined directly from those. If the fluorophores have not been previously characterized or if the fluorescence is expected to be significantly different due to the local environment within a particular cell type, this parameter can be determined experimentally using two cell cultures expressing only the donor and only the acceptor fluorophores. Four spectra are required (donor only cells at λ_{ex2}, donor only cells at λ_{ex1}, acceptor only cells at λ_{ex1}, and acceptor only cells at λ_{ex2}).

$$\begin{aligned}\gamma &= \frac{F_D(\lambda_{em},\lambda_{ex2}) * F_A(\lambda_{em},\lambda_{ex1})}{F_D(\lambda_{em},\lambda_{ex1}) * F_A(\lambda_{em},\lambda_{ex2})}\\ &= \frac{I(\lambda_{ex2}) * [D_{ref}] * s_D * \hat{\varepsilon}_D(\lambda_{ex2}) * Q_D * \hat{e}_D(\lambda_{em}) * I(\lambda_{ex1}) * [A_{ref}] * s_A * \hat{\varepsilon}_A(\lambda_{ex1}) * Q_A * \hat{e}_A(\lambda_{em})}{I(\lambda_{ex1}) * [D_{ref}] * s_D * \hat{\varepsilon}_D(\lambda_{ex1}) * Q_D * \hat{e}_D(\lambda_{em}) * I(\lambda_{ex2}) * [A_{ref}] * s_A * \hat{\varepsilon}_A(\lambda_{ex2}) * Q_A * \hat{e}_A(\lambda_{em})}\\ &= \frac{\hat{\varepsilon}_D(\lambda_{ex2}) * \hat{\varepsilon}_A(\lambda_{ex1})}{\hat{\varepsilon}_D(\lambda_{ex1}) * \hat{\varepsilon}_A(\lambda_{ex2})}\end{aligned} \tag{S10}$$

In order for the intensity and concentration terms to cancel out as shown above, it is important to maintain some of the experimental parameters between certain images for this calibration. The laser power and gain settings must be maintained so that $I(\lambda_{exi})$ for the donor is the same as $I(\lambda_{exi})$ for the acceptor. Additionally, it is important that the images are in perfect registration so that the $[D_{ref}]$ or $[A_{ref}]$ term is constant at both excitation wavelengths for any given pixel. For the Cerulean Venus FRET construct, the γ term determined from literature[20] was 0.045 while the γ term from the above calibration in 3T3 cells was 0.069 (Supplemental Figure S1. This yields a change in the measured C32V FRET efficiency of 0.6% FRET, which is much smaller than the experimental variation due to noise even with significant blurring.

Using the definitions for α, β, and γ we are able to write the magnitude of the acceptor direct excitation in terms of these parameters and correct the acceptor component magnitude such that only the contribution due to FRET is included:

$$\begin{aligned}\text{FRET Acceptor Direct Excitation}(\lambda_{ex2}) + \text{Unpaired Acceptor}(\lambda_{ex2}) &= \frac{\beta}{(1-\gamma)}\\ &\mathit{and}\\ \text{FRET Acceptor Direct Excitation}(\lambda_{ex1}) + \text{Unpaired Acceptor}(\lambda_{ex1}) &= \frac{\beta}{\alpha(\gamma^{-1}-1)}\end{aligned} \tag{S11}$$

$$\begin{aligned}F_{DAcorr}(\lambda_{em},\lambda_{exi}) =& F_{DA}(\lambda_{em},\lambda_{exi}) - \text{Acceptor Direct Excitation}(\lambda_{exi}) + \text{Unpaired Acceptor}(\lambda_{exi})\\ =& I(\lambda_{exi}) * [DA] * s_D * \hat{\varepsilon}_D(\lambda_{exi})\big((1-E) * Q_D * \hat{e}_D(\lambda_{em}) + E * Q_A * \hat{e}_A(\lambda_{em})\big)\\ &+ I(\lambda_{exi}) * [D] * s_D * \hat{\varepsilon}_D(\lambda_{exi}) * Q_D * \hat{e}_D(\lambda_{em})\end{aligned} \tag{S12}$$

The corrected FRET spectra at either frequency can then be unmixed and rearranged to solve for the FRET efficiency, E:

$$\begin{aligned}F_{DAcorr}(donor,\lambda_{exi}) &= F_{DA}(donor,\lambda_{exi})\\ &= I(\lambda_{exi}) * s_D * \hat{\varepsilon}_D(\lambda_{exi}) * Q_D\big([DA] * (1-E) + [D]\big)\\ F_{DAcorr}(acceptor,\lambda_{exi}) &= I(\lambda_{exi}) * [DA] * s_D * \hat{\varepsilon}_D(\lambda_{exi}) * E * Q_A\\ & if [D] \approx 0\\ E(\lambda_{exi}) &= \left(\frac{F_{DAcorr}(donor,\lambda_{exi}) * Q_A}{F_{DAcorr}(acceptor,\lambda_{exi}) * Q_D} + 1\right)^{-1}\end{aligned} \tag{S13}$$

Impact of Instrument Response Function

One of the major concerns with using a literature based calibration is that the detector sensitivity as a function of emission wavelength (also known as the detector's instrument response function, or IRF), will affect the measurement. Differences in the IRF will increase or decrease some of the spectral detector's channels and in turn change the apparent emission shape of the fluorophores, accounting for some of the differences seen between measured and literature spectra, as shown in Supplementary Figure S8. Therefore in the following section we describe how an IRF will manifest itself in the SensorFRET analysis.

Mathematically, an IRF, can be described as an emission wavelength dependent vector (IRF(λ_{em})) being multiplied by each of the experimentally determined spectra as shown below.

$$\begin{aligned}
F_{DA}^{measured}(\lambda_{em},\lambda_{ex}) &= IRF(\lambda_{em}) * F_{DA}^{real}(\lambda_{em},\lambda_{ex}) \\
F_{D}^{measured}(\lambda_{em},\lambda_{ex}) &= IRF(\lambda_{em}) * F_{D}^{real}(\lambda_{em},\lambda_{ex}) \\
F_{A}^{measured}(\lambda_{em},\lambda_{ex}) &= IRF(\lambda_{em}) * F_{A}^{real}(\lambda_{em},\lambda_{ex})
\end{aligned} \tag{S14}$$

Distributing the IRF vector

$$\begin{aligned}
F_{DA}^{measured}(\lambda_{em},\lambda_{ex}) &= I(\lambda_{ex}) * [DA] \Big[s_D * \hat{\varepsilon}_D(\lambda_{ex}) \big((1-E) * Q_D * (IRF(\lambda_{em}) * \hat{e}_D^{real}(\lambda_{em})) + \\
&\quad E * Q_A * (IRF(\lambda_{em}) * \hat{e}_A^{real}(\lambda_{em})) \big) + s_A * \hat{\varepsilon}_A(\lambda_{ex}) * Q_A * (IRF(\lambda_{em}) * \hat{e}_A^{real}(\lambda_{em})) \Big] \\
F_{D}^{measured}(\lambda_{em},\lambda_{ex}) &= I(\lambda_{ex}) * [D] * s_D * \hat{\varepsilon}_D(\lambda_{ex}) * Q_D * (IRF(\lambda_{em}) * \hat{e}_D^{real}(\lambda_{em})) \\
F_{A}^{measured}(\lambda_{em},\lambda_{ex}) &= I(\lambda_{ex}) * [A] * s_A * \hat{\varepsilon}_A(\lambda_{ex}) * Q_A * (IRF(\lambda_{em}) * \hat{e}_A^{real}(\lambda_{em}))
\end{aligned} \tag{S15}$$

Making the substitutions $\hat{e}_D^{measured} = IRF(\lambda_{em}) * \hat{e}_D^{real}$ and $\hat{e}_A^{measured} = IRF(\lambda_{em}) * \hat{e}_A^{real}$, we can see that unmixing $F_{DA}^{measured}(\lambda_{em},\lambda_{ex})$ with $\hat{e}_D^{measured}$ and $\hat{e}_A^{measured}$ provides the same exact expressions as shown in equation S4. This implies that if the component shapes are measured using the same instrument (and therefore the same IRF), the unmixing results are identical to a perfectly calibrated detector (IRF=1 for all wavelengths). Of course if the IRF associated with a literature emission spectra is different than the IRF associated with the measured FRET spectra, the component magnitudes determined by the unmixing and therefore the measured FRET efficiency will be affected.

The other aspect of the analysis that may be affected by the IRF is the determination of the γ parameter. As the IRF vector is independent of excitation frequency, when taking the ratios $\frac{F_D^{measured}(\lambda_{em},\lambda_{ex2})}{F_D^{measured}(\lambda_{em},\lambda_{ex1})}$ and $\frac{F_A^{measured}(\lambda_{em},\lambda_{ex1})}{F_A^{measured}(\lambda_{em},\lambda_{ex2})}$, the IRF is canceled out, yielding the exact same ratios as $\frac{F_D^{real}(\lambda_{em},\lambda_{ex2})}{F_D^{real}(\lambda_{em},\lambda_{ex1})}$ and $\frac{F_A^{real}(\lambda_{em},\lambda_{ex1})}{F_A^{real}(\lambda_{em},\lambda_{ex2})}$. This in turn implies that the measured γ parameters are valid even on equipment with significantly different IRF. Therefore, the IRF cannot affect any of the parameters used to calculate the FRET efficiency if an experimental calibration is performed and only the normalized emission spectra aspect of the literature based calibration could be rendered invalid by differences in the IRF.

Simulation Dataset Structure

The simulation dataset consists of 10 components used in our simulations. The matlab file can be read in with each of the components as a separate variable. The python numpy array can be read in and then converted to a dictionary containing each component using numpy.load('YourFilepath').items().

Matlab Variable/Dictionary Entry: 'Simulated Pixels': These are simulated noisy spectra covering a range of SNR and FRET efficiencies, organized in the following manner- Simulated Pixels [N,Power,Efficiency,Excitation,Emission], where N are repeat simulations to calculate statistics with (1000 simulations for every condition). Has an overall shape of (1000, 150, 11, 2, 32).

'sRET/luxFRET Calibration Spectra': These are noiseless calibration spectra organized in the following manner- Calibration Spectra [Power,Donor or Acceptor,Excitation,Emission], with an overall shape of (150, 2, 2, 32). These spectra can also be used to calculate the normalized emission spectra and gamma parameter needed for sensorFRET, but we also included those values separately for convenience.

'Power Vector': The vector relating the indices in the 2nd dimension of 'Simulated Pixels' to the simulated power used ranging from 0.1-1000 (arbitrary units) in 150 logarithmic steps to change the SNR and provide normalized residuals in the approximate range of 0.001 to 0.1.

'Efficiency Vector': The vector relating the indices in the 3rd dimension of 'Simulated Pixels' to the simulated FRET efficiency ranging from 0 to 1 in 11 linear steps.

'Excitation Wavelength Vector': The vector relating the indices of the 4th dimension of 'Simulated Pixels' to the simulated Excitation wavelength, either 405 or 458.

'Emission Wavelength Vector': The vector relating the indices of the 5th dimension of 'Simulated Pixels' to the simulated Emission Wavelengths, ranging from 416 to 718 in 32 linear steps to match the spectral resolution of our experiments.

'Normalized Emission Spectra':An array containing the normalized emission shapes for the Cerulean and Venus fluorophores (shape of (2, 32)).

’Gamma’: the sensorFRET gamma parameter for the Cerulean/Venus-405/458 pairing, 0.0605 (from experiment)
’Qd’: the quantum efficiency of Cerulean, 0.62.
’Qa’: the quantum efficiency of Venus, 0.57.

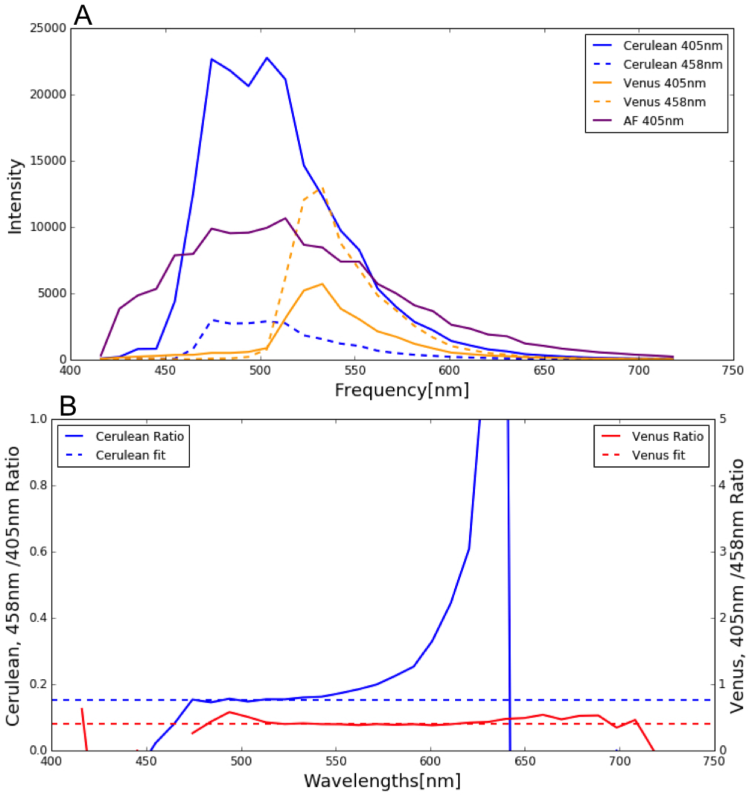

Figure S1. Calibration measurements for Cerulean and Venus: A) Background-subtracted single fluorophore measured emission spectra at 405nm and 458nm excitation frequencies. Emission spectra are computed from an average of all pixels in the calibration images with saturated pixels excluded. The purple autofluorescent component was measured on an unlabeled sample at 405nm excitation with the laser power set to peak value to maximize the SNR. B) Cerulean (blue) and Venus (red) spectra ratios used to compute gamma. Taking the product of the dotted red and blue lines outputs the gamma term. The dotted lines are determined by least squares fitting of the spectra in A using the normalized emission shapes determined from 458nm measurements of Cerulean-alone and Venus-alone samples.

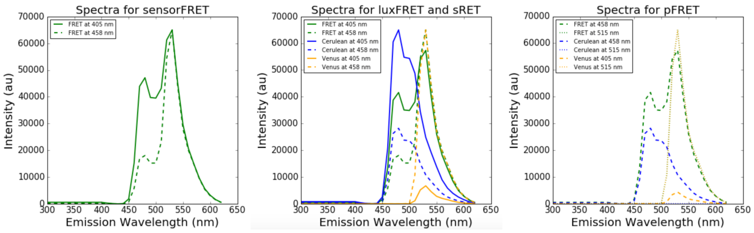

Figure S2. Noiseless spectra required for SensorFRET, luxFRET, sRET, and pFRET analysis approaches simulated using normalized excitation spectra, emission spectra, and quantum efficiencies from literature[23]

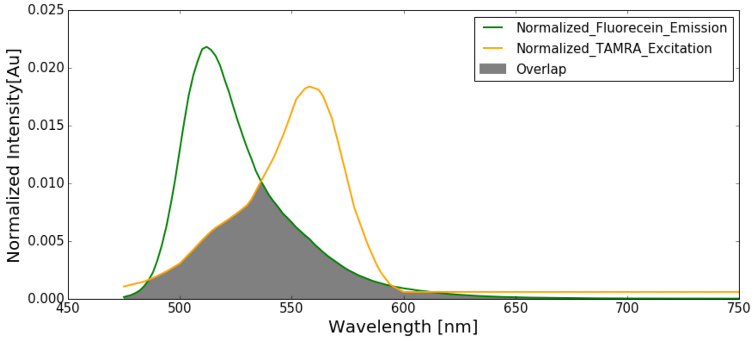

Figure S3. Overlap between of Fluorecein emission and TAMRA excitation spectra showing the pair is suitable for FRET.

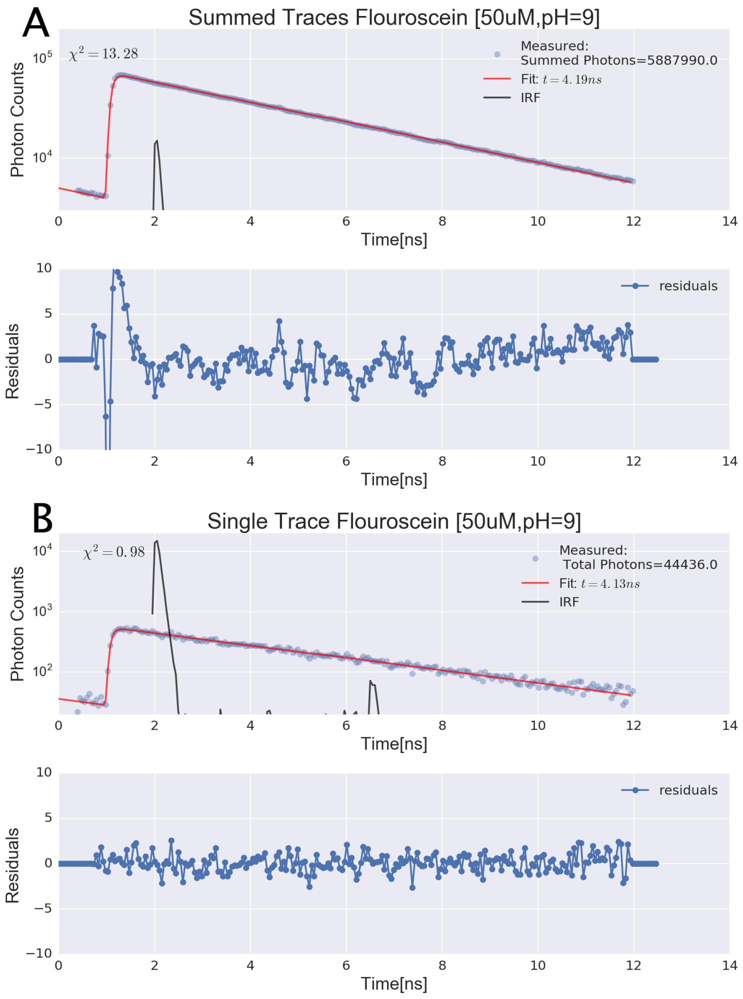

Figure S4. Demonstration of TCSPC Calibration: 50μM Fluorescein fluorescent lifetime decays fittings in sodium borate (pH-9). A) global (256x256) or B) local (10x10) binning of lifetime images were used to aggregate photon counts into two separate decay traces (Photon Counts:N_A=5.8E6 and N_B=4.4E5). Non-linear least squares fitting of a single exponential decay model (red) yielded a decay constant of A) 4.19ns or B) 4.13ns. The measured instrumental response function (IRF) is shown in black and the goodness of fit (χ^2 statistic) measured A) 13.28 and B) 0.98 respectively. Fit residuals are plotted below each decay trace in blue.

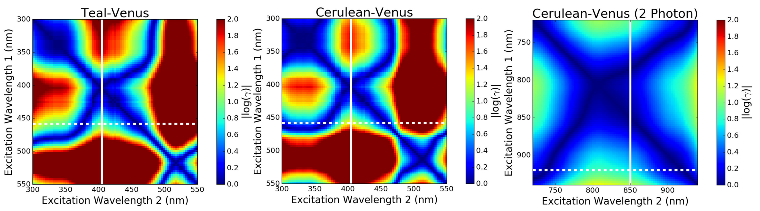

Figure S5. Calculated γ constant for Teal-Venus and Cerulean-Venus at all possible excitation pairings. Intersection of the white lines correspond to the excitation frequencies used in the experiments, showing that γ at these pairings do not equal 1 and are therefore suitable for FRET analysis.

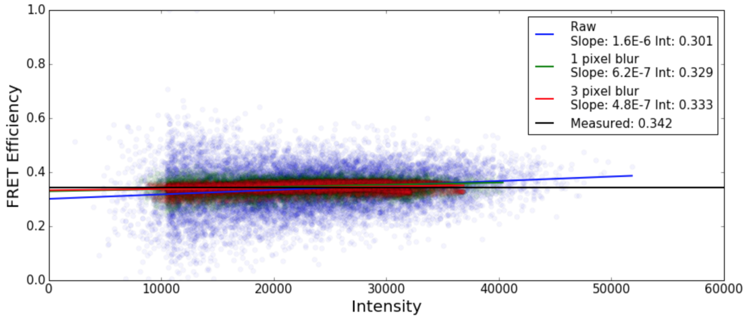

Figure S6. Scatterplot of measured FRET vs Intensity for the C32V FRET standard at varying Gaussian blur sizes. Increasing FRET efficiency with increasing intensity can be indicative of intermolecular FRET.

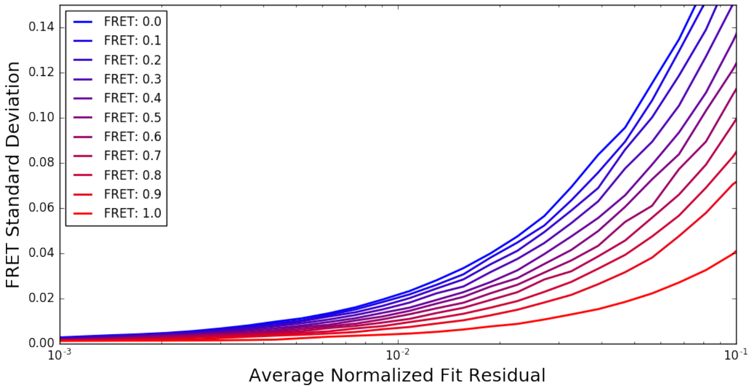

Figure S7. Series of simulated FRET standard deviation vs Normalized Fit Residual curves for 0.1 FRET efficiency steps in mTFP-Venus constructs. At each simulated FRET efficiency, 1000 pixels were used to compute the standard deviation and the average normalized residual of the least squares fit. To generate each curve, Equation S2 was used and Poisson noise was added based on the noise model shown in Figure 2

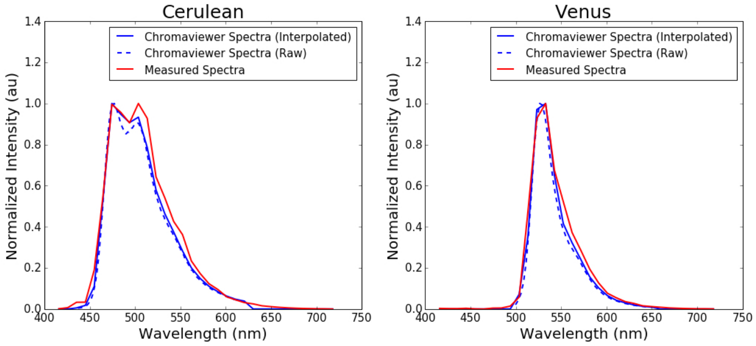

Figure S8. Comparison of Cerulean and Venus emission spectra determined experimentally or obtained through literature. Small differences can be observed which are due to differences in either the instrument response function or environment. To use with experimental FRET spectra, literature spectra must be interpolated to the same number of channels as used in experiment. Raw spectra are averaged over 10nm spectral bands at the channel frequencies used in experiment to obtain the interpolated spectra
